# Synthesis of Some New Folic Acid-Based Heterocycles of Anticipated Biological Activity

**DOI:** 10.3390/molecules26020368

**Published:** 2021-01-12

**Authors:** Ola A. Abu Ali, Hosam A. Saad, Bodor M. A. Al Malki

**Affiliations:** Department of Chemistry, College of Science, Taif University, P.O. Box 11099, Taif 21944, Saudi Arabia; h.saad@tu.edu.sa (H.A.S.); bedoo201044@hotmail.com (B.M.A.A.M.)

**Keywords:** folic acid, glutamic acid, imidazo[2,1-*b*]pteridine, tetrahydroimidazo[2,1-*b*]pteridine, biological activity

## Abstract

To date, no fused heterocycles have been formed on folic acid molecules; for this reason, and others, our target is to synthesize new derivatives of folic acid as isolated or fused systems. Folic acid **1** reacted with ethyl pyruvate, triethyl orthoformate, ethyl chloroformate, thioformic acid hydrazide, and aldehydes to give new derivatives of folic acid **2**–**6a**,**b**. Moreover, It reacted with benzylidene malononitrile, acetylacetone, ninhydrin, ethyl acetoacetate, ethyl cyanoacetate, and ethyl chloroacetate to give the pteridine fused systems **10**–**15**, respectively. Ethoxycarbonylamino derivate **5** reacted with some nucleophiles containing the NH_2_ group, such as aminoguanidinium hydrocarbonate, hydrazine hydrate, glycine, thioformic acid hydrazide, and sulfa drugs in different conditions to give the urea derivatives **16**–**20a**,**b**. Compound **4** reacted with the same nucleophiles to give the methylidene amino derivatives **21**–**24a**,**b**. The fused compound **10** reacted with thioglycolic acid carbon disulfide, malononitrile, and formamide to give the four cyclic fused systems **25**–**30**, respectively. The biological activity of some synthesized showed moderate effect against bacteria, but no effect shown towards fungi.

## 1. Introduction

Folic acid **1** is essential to the human metabolic process. It is a key factor in the synthesis of nucleic acid. Folic acid (vitamin B_9_) helps with growth [1] and healthy red blood cells (RBCs) [2]. It is important for cell division. It is essential for growth of the fetus [3]. Folic acid deficiency can lead to human megaloblastic anemia and neural tube defects in fetuses [4], as well as heart diseases, and cancer [5]. To avoid all of these risks, folic acid intake from fortified food has been increasing rapidly [6].

Several novel 2,4-diamino-5-deaza-6,7,8,9-tetrahydropyrido[3,4-*g*]pteridine derivatives with different substitution at the N_7_ position were designed and synthesized, as classical and non-classical, partially restricted, linear tricyclic 5-deaza antifolates. The purpose was to investigate the effect of conformational restriction of the *C*_6_−*C*_9_ (τ1) and *C*_9_−*N*_10_ (τ2) bonds via an ethyl bridge from the *N*_10_ to the *C*_7_ position of 5-deaza methotrexate (MTX) on the inhibitory potency against dihydrofolate reductase (DHFR) from different sources and on antitumor activity [7]. Moreover, some efforts were carried out to synthesize the 7-substituted folic acid derivatives the less toxic, more effective, and selective agents for cancer chemotherapy based upon inhibition of dihydrofolate reductase and thymidylate synthetase [8]. The 10-Alkyl-5,10-dideaza analogs of methotrexate and tetrahydrofolic acid were synthesized and used as potent inhibitors of glycineamide ribonucleotide (GAR) formyltransferase [9]. Moreover, numerous fused cyclopenta[d]pyrimidine antifolate were synthesized and examined for their effects as highly potent as DHFR and cell growth inhibitors, and most of them are more potent than methotrexate (MTX) and 10-ethyl-10-deazapterin (10-EDAM) in inhibiting tumor cell growth (P388 MTX-sensitive and MTX-resistant, colon 26 and KB) on 72 h drug exposure [10]. Furthermore, 5-deaza-7-desmethylene analogues of 5,10-alkylene-5,6,7,8-tetrahydrofolic acid were good substrates for mouse liver folylpolyglutamate synthetase [11].

Bacteria in the intestine synthesizes folic acid [12], which is essential for the progress of the neurological systems of fetuses [13]. Folic acid, as well as the plasma concentration of folate, were inversely associated with hematological, cardiovascular diseases, as well as colorectal cancer [14,15]. Folic acid exhibited anti-inflammatory, antioxidant effects, and decreased levels of interleukins [16,17]. A previous study revealed that folic acid treatment suppressed the inflammatory response of human monocytic cells (THP-1 cells) through the PI3K/Akt pathway [18].

Due to the essential role of folic acid in the synthesis of the bacterial cell wall, we adopted an idea of confusing the bacteria in the synthesis of the cell wall by using a folic acid analogue as an anti-cell wall synthesis drug. Therefore, the target of this work was synthesize some new derivatives from folic acid and screening them against some G+, G− bacteria, and fungi, in the hope of obtaining new antibacterial or antifungal compounds.

**Figure 1:**
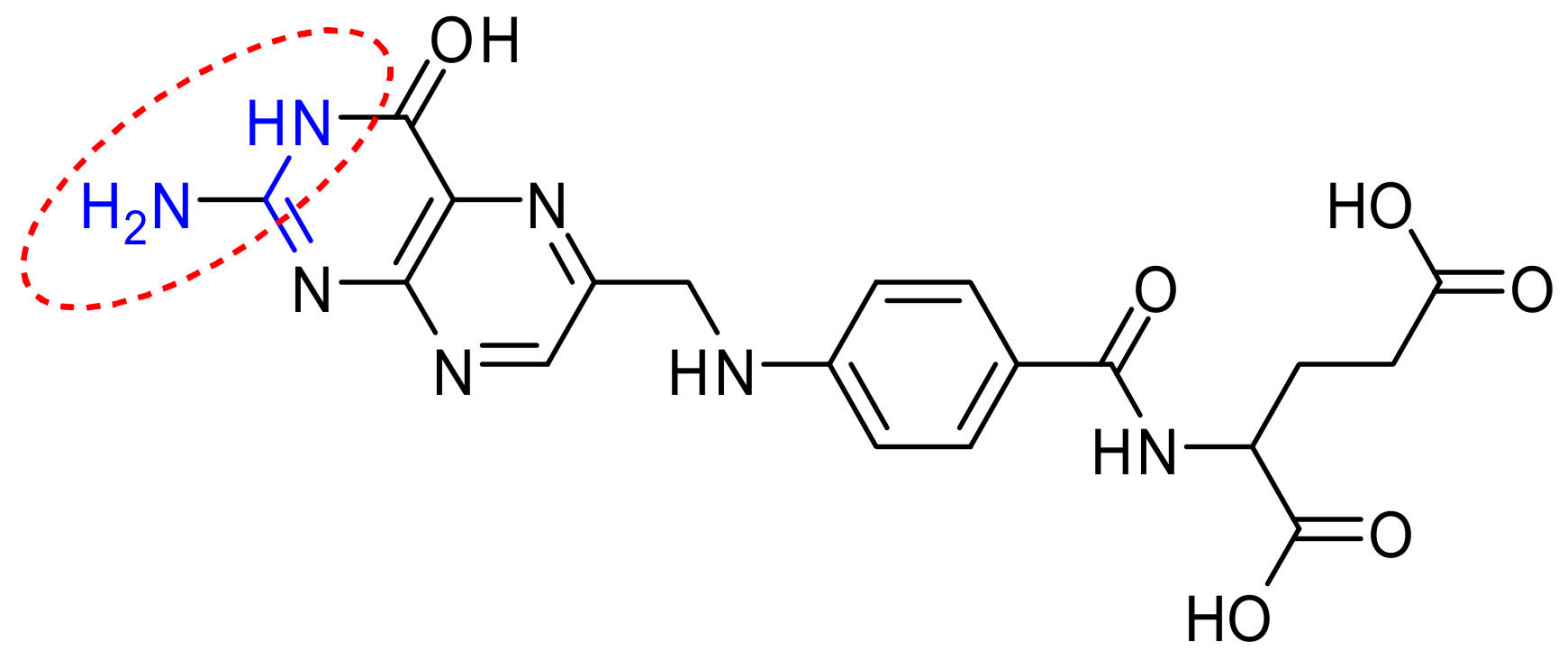
Folic acid reaction center.

The main reaction center of folic acid illustrated in Figure 1.

## 2. Results and Discussion

The importance of synthesizing a new heterocyclic compound is strongly bonded with the finding of a new drug, capable of destroying any of the diseases that spread around the world. Herein is a new trial, in continuation of our previous work [19,20,21,22,23,24,25,26,27,28,29,30,31,32,33,34,35,36,37,38,39,40,41,42,43], to synthesize a new compound from folic acid in the hope of getting a new promoting drug.

**Scheme 1:**
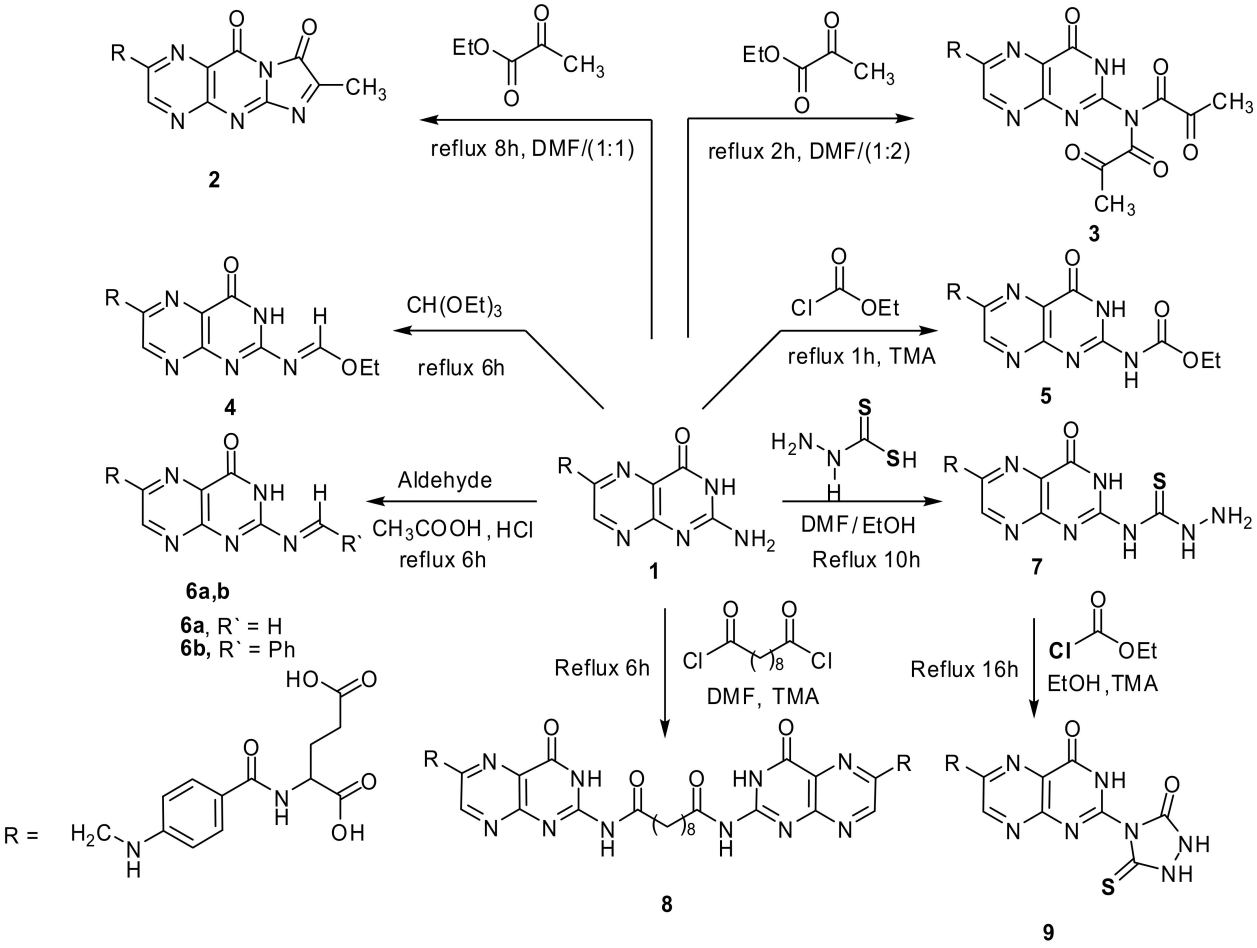
Synthesis some new folic acid derivatives.

Folic acid **1** reacted with ethyl pyruvate in different ratios in DMF as a solvent. Refluxing mixture of folic acid and ethyl pyruvate 1:1 ratio for 8 hr, gave imidazo[2,1-*b*]pteridine derivative **2**. While the reaction of folic acid with ethyl pyruvate, in a 1:2 ratio for 2 hr, led to the formation of the *N*,*N*-disubstituted folic acid (Scheme 1). The structures of the two compounds **2** and **3** were confirmed from their IR and NMR spectra, where in the IR spectra, the band due to the NH_2_ group disappeared with appearance of new bands due to the inserted carbonyl groups in the two compounds. While, ^1^H-NMR of compound **2** showed singlet signal at δ = 1.30 ppm due to the CH_3_ attached to the imidazole ring. The ^1^H-NMR of compound **3** also showed more deshielded singlet signal at δ = 2.21 ppm due to the 2CH_3_ attached to the carbonyl group. The ^13^C-NMR supported the structure proposed where it showed signals due to the CH_3_ in the two compounds at δ = 24.51 and 26.79 ppm, respectively.

Formation of ethoxymethyleneaminopetridine derivative **4** and ethoxycarbonylaminopetridine derivative **5** were formed by the reaction of folic acid with excess triethyl orthoformate and/or ethyl chloroformate in presence of TMA as a base (Scheme 1). The elucidation of the structures of the two compounds were recognized from their spectra and elemental analysis where, the two compounds showed the appearance of new signals in the ^1^H-NMR, triplets at δ = 1.15 and 1.14 quartets at 3.39 and 4.02 ppm due to the CH_3_ and CH_2_, respectively. The condensation of the NH_2_ of folic acid with aldehyde to form Schiff base was carried using formaldehyde and benzaldehyde in acetic acid glacial using drops of HCl as an acidic catalyst. The Schiff bases formed **6a**,**b** showed the disappearance of the NH_2_ group in folic acid and appearance for the olefinic proton due to the formation of N=CH bond at δ = 7.99 and 8.03 ppm, respectively. ^13^C-NMR helped in the structures conformation, where, it showed signals at δ = 139.75 and 164.21 ppm due to the methylidene and benzylidene carbons respectively. The reaction of thioformic acid hydrazide with folic acid in DMF/EtOH mixture yielded the thiosemicarbazide derivative **7** which cyclized with ethyl chloroformate in presence of TMA to give the 1,3,4-triazole thione derivative **9** (Scheme 1). The structures of compounds **7** and **9** were established from IR and ^1^HNMR, where the IR showed extra bands due to the NH groups in compound **7** and two bands due to C=S at 1338 and 1347 cm^-1^ for compounds **7** and **9**, respectively. Moreover, ^1^H-NMR showed three singlet signals for the thiosemicarbazide group at 5.69 (NH_2_), 11.31 and 12.89 ppm for (NHCSNH) protons respectively. Folic acid, also, reacted with acid chloride derivative in basic medium to give the *N*-substituted derivative. Reaction of folic acid with sebacoyl chloride in presence of TMA yielded the bis compound **8** (Scheme 1). The ^1^H-NMR confirmed the structure of **8** where it showed characteristic signals for the 8 CH_2_ due to the sebacoyl moiety at δ = 1.03, 1.26, 2.23, and 3.34 ppm, respectively.

**Scheme 2:**
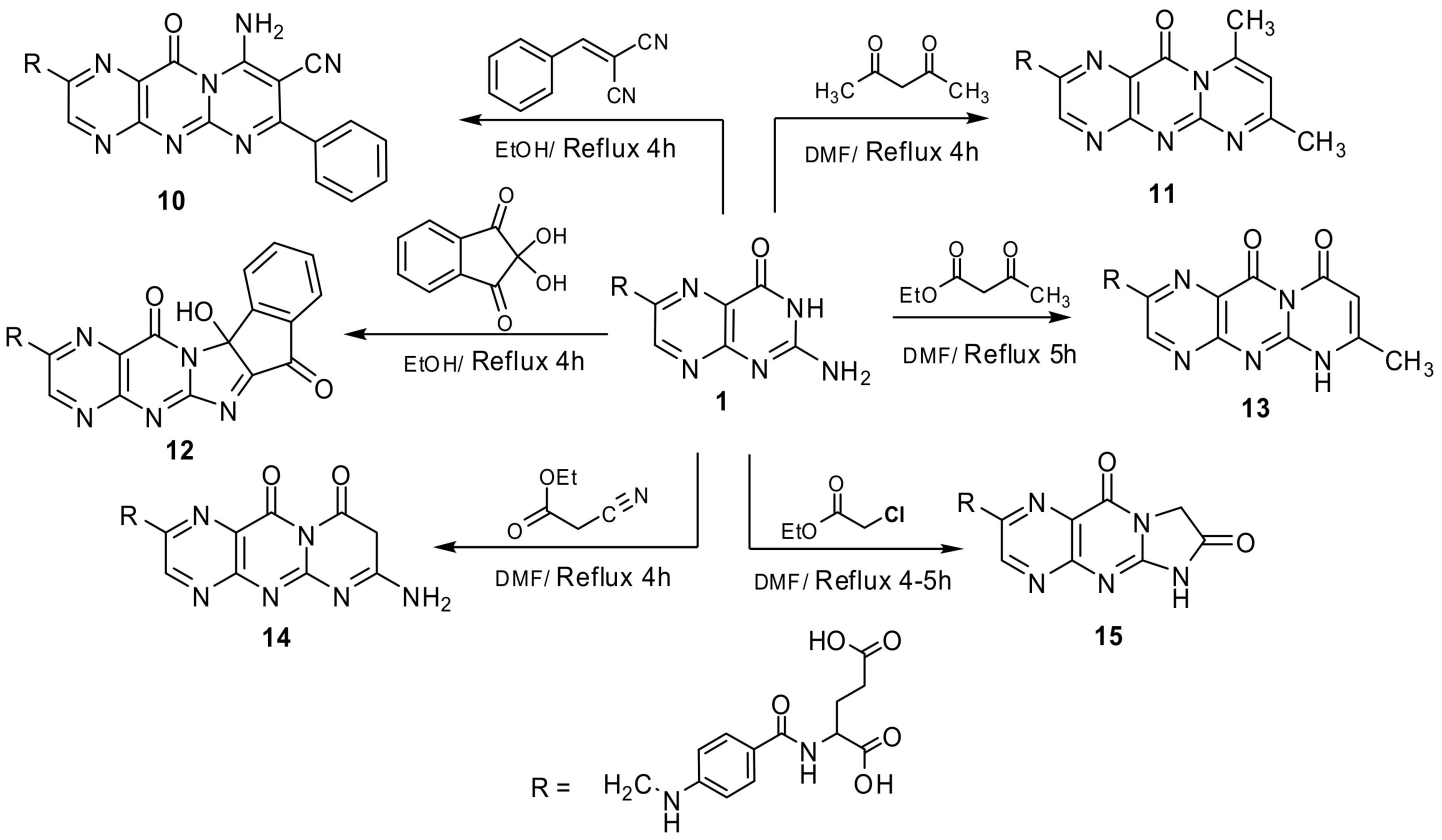
Synthesis of some new fused systems built on folic acid.

Fused systems built in folic acid molecule another target for discovering new promising drug. (Scheme 2), showed some reactions resulted in the formation of fused systems with three or five fused rings.

Benzylidene malononitrile was the first compound was used to form the nucleus compound for synthesizing the more fused rings. The well-known benzylidene malononitrile was synthesized and reacted with folic acid in boiling ethanol to form the three fused rings compound pyrimido[2,1-b]pteridine derivative **10** (Scheme 2) The appearance of the CN band in the IR of compound **10** was the first guide for the structure assertion. The ^1^H-NMR was the second guide, where it showed multiplets at δ = 7.62–7.96 ppm for the 7-phenyl group. Reaction of folic acid with acetylacetone, ethyl acetoacetate, ethyl cyanoacetate, and/or ethyl chloroacetate in DMF gave fused pyrimidine, fused primidone and fused imidazolidinone to the pteridine ring **11**, **13**, **14,** and **15**, respectively (Scheme 2). While the reaction of folic acid with ninhydrin in boiling ethanol yielded the five fused rings derivative indeno[2′,1′:4,5]imidazo[2,1-b]pteridine **12**. The structures of the resulted compounds stablished from their ^1^H-NMR and ^13^C-NMR. The ^1^H-NMR of compound **11** showed two singlets at δ = 2.26 and 2.40 ppm for the two CH_3_- in pyrimidine ring, Moreover, two signals for the same groups appeared at 24.30 and 26.45 ppm in the ^13^C-NMR. Compound **13** showed two singlets one for the CH_3_ at 2.25 and the other at 7.21 due to pyrimidone olefinic proton. The structure of compound **14** was confirmed by the singlet appeared in its ^1^H-NMR at δ = 2.89 ppm due to pyrimidone CH_2_, which also appeared in its ^13^C-NMR at δ = 43.05 ppm. The disappearance of the CN band for compound **14** in its IR proved the cyclic structure of the compound. The ^1^H-NMR of compound **15** was the main guide for its structure proven, where the ^1^H-NMR of compound **15** showed singlet at δ = 3.51 ppm due to imidazolidinone CH_2_ along with the presence of NH singlet signal of imidazolidinone at δ = 10.87 ppm. On the other hand, the structure of compound **12** was certain, by the presence of the singlet due to the imidazoloindene OH group at δ = 5.36 ppm.

**Scheme 3:**
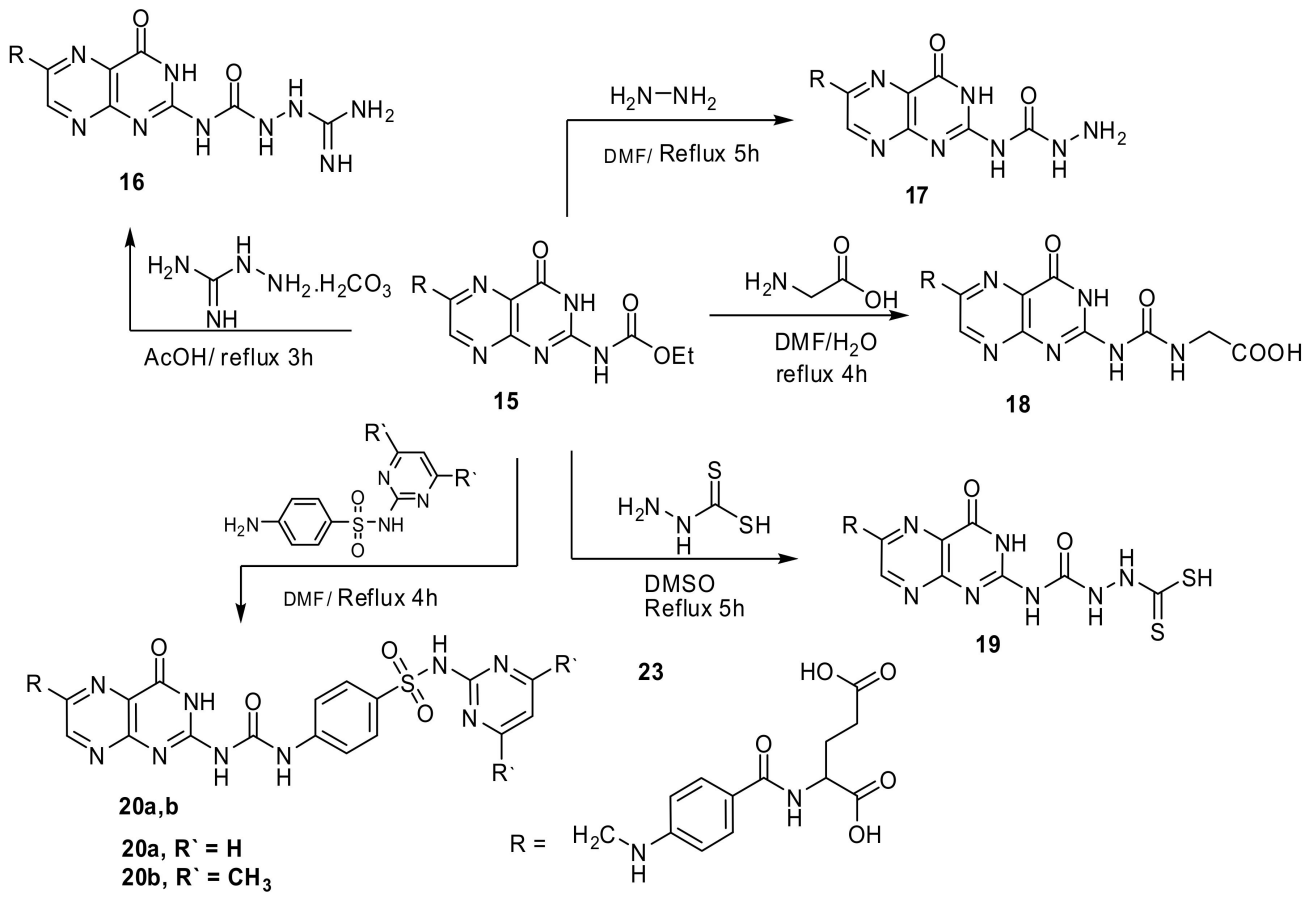
Synthesis of some urea derivatives substituted on folic acid.

Ethoxycarbonylamino derivative **15** was used as starting material for the synthesis of other types of derivatives, which are not attached directly to the pteridine ring. We exploited the chance presence of an ester group in the compound and subjected it to react with some nucleophiles containing NH_2_, such as aminoguanidinium hydrocarbonate, hydrazine hydrate, glycine, thioformic acid hydrazide, and sulfa drugs in different conditions. Reaction of compound **15** with aminoguanidinium hydrocarbonate in boiling glacial acetic acid yielded “*N*-{4-[({2-[({2-[amino(imino)methyl]hydrazino}-carbonyl)amino]-4-oxo-3,4-dihydropteridin-6-yl}methyl)amino]benzoyl}-glutamic acid” **16** (Scheme 3). The structure of **16** was elucidated from the disappearance of the two signals characteristic for the ethyl group in its ^1^H-NMR, with appearance of multi signals for different NH present in the compound (experimental part).

Reaction of **15** with NH_2_NH_2_ in boiling DMF yielded the semicarbazide derivative **17**. Reaction of **15** with glycine in DMF/H2O mixture resulted in the carboxymethylurea derivative **18**. Thioformic acid also reacted with compound **15** in boiling DMSO and gave thiocarboxysemicarbazide derivative **19** (Scheme 3). Conformation of **17**, **18**, and **19** structures were elucidated form their spectral and analysis data, where all of the ^1^H-NMR spectra of all the compounds missing the ethyl group, triplet and quartet signals, with appearance of other signals due to the new functional groups, for example, compound 126, showed in its ^1^H-NMR signals at δ = 4.33, 8.69 and 9.61 ppm for the NH_2_, and two NH proton neighboring to the carbonyl group. Compound **18** showed the signals corresponding to the glycine molecule, the CH_2_ protons appeared at δ = 4.03 ppm and the acidic proton of the glycine carboxylic group found at 11.60 ppm. The ^13^C-NMR of compound **18** also supported the structure elucidation, where it showed a signal for CH_2_ carbon at 41.10 ppm and another signal for the glycine carboxylic carbon at 173.17 ppm. Compound **19** was the highest data help in the structure confirmation, where, Its IR showed bands due to thiocarboxylic group at 2627 cm^−1^ for (SH) and 1351 cm^−1^ for (C=S) group. Th ^1^H-NMR of compound **19** gave more conformational data, it showed signal due to the SH group at δ = 13.98 ppm, all of this data are supported with the elemental analysis for the sulfur. The presence of the SH group in compound **19** rejected the idea that compound **19** may cyclize to give 1,3,4-triazole thione derivative.

The combination between the sulfa drug and folic acid was an idea for increasing the biological activity of folic acid, especially antibacterial activity. Thus, folic acid reacted with sulfadiazine and/or sulfadimidine in DMF to yield the corresponding aminocarbonyl sulfadiazine derivative **20a** and aminocarbonyl sulfadimidine derivative **20b**, respectively (Scheme 3). The structures of **20a** and **20b** were confirmed from their spectral data (experimental part].

**Scheme 4:**
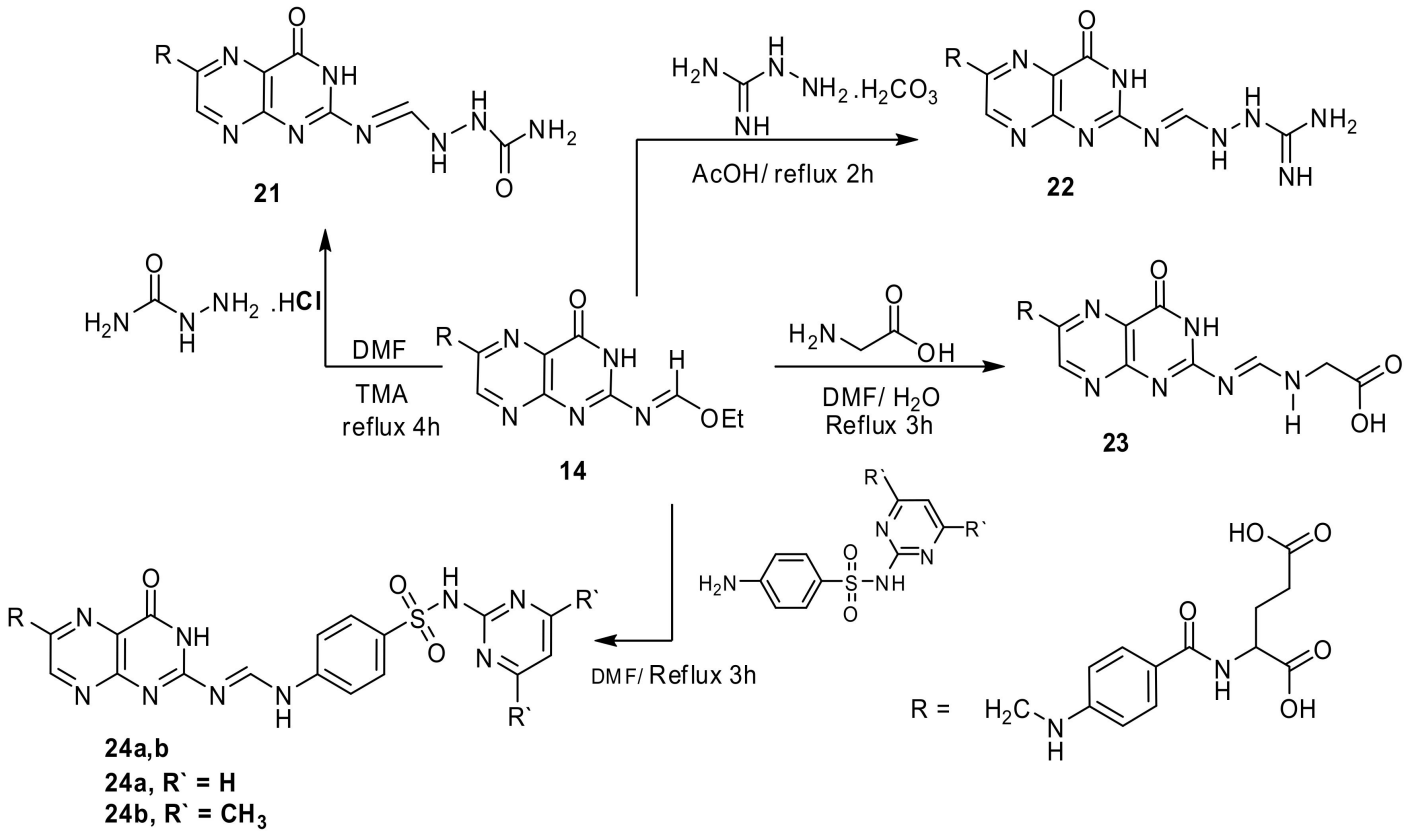
Synthesis of some methylene amino derivatives substituted on folic acid.

Ethoxymethylene derivative **14** was used for synthesizing a new folic acid derivative; thus, compound 113 reacted with some nucleophilic amino compounds, such as, semicarbazide hydrochloride, aminoguanidine hydrocarbonate, glycine, and sulfa drugs, to obtain some new Schiff base derivatives. Reaction of **4** with semicarbazide hydrochloride in boiling DMF in presence of drops of TMA as a base yielded “*N*-[4-({[2-({[2-(aminocarbonyl)hydrazino]methylene}amino)-4-oxo-3,4-dihydropteridin-6-yl]methyl}-amino)benzoyl]glutamic acid” **21**, while the reaction with aminoguanidine hydrocarbonate in glacial acetic acid yielded the “*N*-{4-[({2-[({2-[amino(imino)methyl]-hydrazino}methylene)amino]-4-oxo-3,4-dihydropteridin-6-yl}methyl)amino]benzoyl}-glutamic acid” **22**. Moreover, reaction of **14** with glycine in mixture of DMF/ H2O (9:1) gave the carboxymethyl)amino derivative **23** (Scheme 4). The structure of the formed compounds **21**, **22,** and **23** are confirmed from their IR, ^1^H-NMR, and ^13^C-NMR, where all compounds showed the disappearance of the two signals of the ethyl group in their ^1^H-NMR with appearance of new signals due to NH_2_, and NH groups at the ranges 6.21–6.98 ppm for NH2 group and 10.13–10.99 ppm for NH groups. Compound **23** showed in its ^1^H-NMR two signals for the glycine moiety, at singlet at δ = 4.18 ppm for CH_2_ and at 11.58 for the acidic proton CH_2_COOH. Compound **14** reacted with sulfa drugs, namely, sulfadiazine and sulfadimidine, to give the folic acid substituted with sulfa drug moiety, **24a**,**b** (Scheme 4). The structures of compounds **24a**,**b** proved with the aid of their ^1^H-NMR, where, the ^1^H-NMR showed the appearance of the multiplets due to the phenyl group of the sulfa drug, with disappearance of the ethyl group signals.

**Scheme 5:**
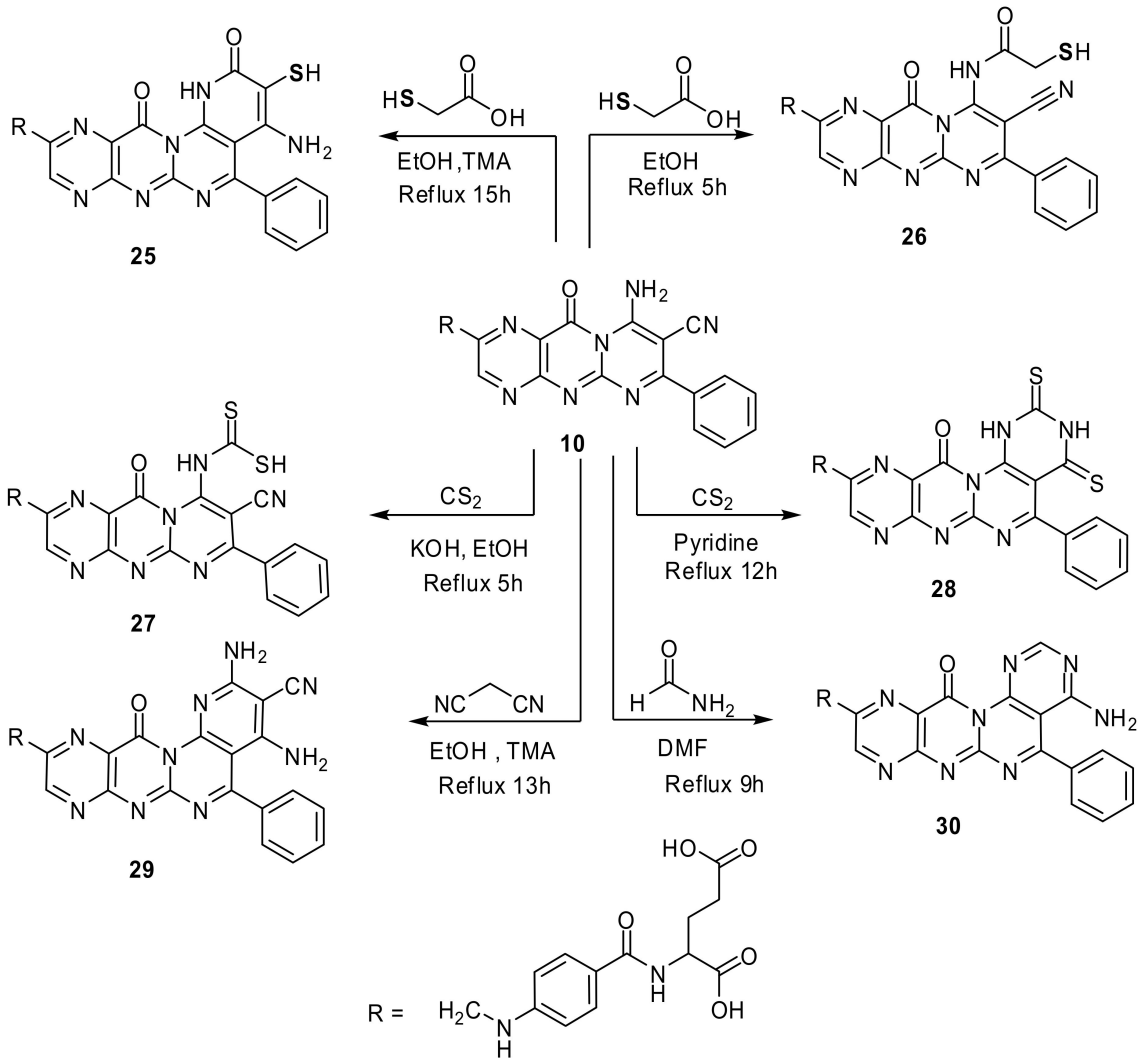
Synthesis of some new fused systems on folic acid.

Finally, compound **10** was used for building some new fused systems on the folic acid molecule. It subjected for some reactions, which gave four ring fused systems as a new form for folic acid derivatives. Compound **10** reacted with thioglycolic acid in basic conditions to yield the tetracyclic fused system “*N*-(4-{[(4-amino-3-mercapto-2,12-dioxo-5-phenyl-1,12-dihydro-2*H*-pyrido-[3′,2′:5,6]pyrimido[2,1-*b*]pteridin-10-yl)methyl]-amino}benzoyl)-glutamic acid” **25** (Scheme 5). Reaction of **10** with thioglycolic acid boiling ethanol without using TMA yielded the open form mercaptoacetyl derivative **26** (Scheme 5). The two structures of **25** and **26** in the ^1^H-NMR showed the presence of the SH group at 14.00 and 13.97 ppm, respectively. The disappearance of the CN group in the IR of compound **25** confirmed the cyclic form of the compound, while its appearance in the IR of **26** prove that the structure is an open form.

The reaction of carbon sulfide with compound **10** varies with the kind of solvent used, whereas the reaction of **10** with CS_2_ in alcoholic KOH yielded the open form N-{4-[({8-cyano-9-[(mercaptocarbonothioyl)amino]-11-oxo-7-phenyl-11H-pyrimido[2,1-b]pteridin-2-yl}methyl)amino]-benzoyl}glutamic acid **27**, while the reaction in boiling dry pyridine yielded the four cyclic ring fused system **28** (Scheme 5). The IR of the two compounds helped in elucidation of the structures, where the IR of compound **27** showed CN band at 2202 cm^−1^, which are not present in the IR of compound **28**; this is an indication that compound **27** is open form and **28** is a cyclic form. Pyrido[3′,2′:5,6] pyrimido[2,1-b]pteridine **29** and pyrimido[5′,4′:5,6]pyrimido-[2,1-*b*]pteridine **30** were prepared by the reaction of **10** with malononitrile and/or formamide on hot. Compound **29** showed, in its ^1^H-NMR, two signals for the two NH_2_ groups at δ = 6.12 and 6.58 ppm, while compound **30** showed only one signal for the NH_2_ groups at δ = 6.50 ppm.

## 3. Biological Activity

Antimicrobial activities of some new compounds were tested against some types of G^+^ bacteria, G^−^ bacteria, and fungus, for example, “*Bacillus subtilis* (ATCC, 6051), *Staphylococcus aureus* (ATCC, 12600), as a G^+^ bacteria, *Escherichia coli* (ATCC, 11775), *Pseudomonas aeruginosa* (ATCC, 10145), as a G^−^ bacteria, *Aspergillus flavus* link (ATCC, 9643), and *Candida albicans* (ATCC, 7102), as a fungus”. Antimicrobial activity of the tested samples was determined using a modified Kirby–Bauer disc diffusion method [44,45,46,47,48,49].

### Biological Activity Results and Discussion

The studying of the biological screening of the tested compounds, Table 1, showed folic acid had a moderate inhibitory effect against each of the G^+^ and G^−^ bacteria compared to the standard antibacterial agent (Ampicillin).

Compounds **6a**, **8**, **22**, **26,** and **27** showed inhibitory effects slightly more than folic acid against all types of bacteria, and this may be due to the presence a new group exceeded to folic acid structure, such as, terminal C=N in compounds **6a** and **22**, SH in compounds **26** and **27**, and 8 CH_2_ in compound **8**.

Inden-1-one present in compound **12** may be the cause for more activity than folic acid against G^−^ bacteria *E*. *coli* and *P*. *aeruginosa*.

Compound **9** as well as compound **20b** showed somewhat strange inhibitory effects, where compound **9** showed higher effect against *P*. *aeruginosa* than folic acid, and at the same time, it showed null effects against *E*. *coli* and *S*. *aureus;* this may be due to the presence of triazole thione ring in its structure. Moreover, compound **20b** showed null effects against *B*. *subtilis* and *P*. *aeruginosa* and moderate effect against *E*. *coli* and *S*. *aureus,* the diamidine nucleus in its structure.

Compounds **2**, **4**, **10**, **14**, **15**, **23**, and **24b** showed nearly the same effects similar to folic acid; this means that the new groups added to the structure of folic acid did not have any effect against microorganisms in general.

Compounds **5** and **18** had no effect against all microorganisms; this may be due to the presence of each of glycine unit and/or the ethyl carbamate unit in the structure.

In comparison, among the different effects of all compounds under study, we found:

- Compound **22** showed higher effect against *B*. *subtilis* than other compounds that showed an average effect.
- Compounds **5**, **18**, **20b** did not show the effect against *B*. *subtilis*.

The effect of compounds **6a**, **8**, **22**, **27** was slightly higher than **11**, **12**, **14**, **23** against *S*. *aureus* while other compounds have a moderate effect against *S. aureus* and compounds **1**, **5**, **18** had no effect against them.

The effect of compounds against *E*. *coli* was close to the effect of compounds against bacteria, while the effect of compounds against *P*. *aeruginosa* was dissimilar. Compound **27** showed a higher effect against *P*. *aeruginosa* than other compounds.

On the other hand, folic acid, as well as all of the synthesized compounds, had no effect against Fungi (*A*. *flavus* and *C*. *albicans*).

## 4. Conclusions

Herein, novel derivatives of folic acid were prepared by direct reactions of different reagents, with folic acid, or by reaction of some derivatives prepared with the same (or another) reagent. The study was directed to find new derivatives of folic acid having a promising biological activity, but, from the study carried out and the results obtained, all of the derivatives prepared were inactive against fungi, while some of these derivatives had a moderate antibacterial activity. The conclusion of this study is that the reactions done on the NH_2_ group of folic acid, either substituted groups formed or fused systems, are not valuable as antifungal or antibacterial agents. Future work will be directed to the carboxylic groups of folic acid to synthesize a new isolated or fused system from folic acid in hopes of getting a more promising drug.

### 4.1. Experimental Chemistry

All chemicals used were supplied by Sigma (New York, NY, USA). Digital Electro thermal IA 9100 Series used for measuring melting points and they were uncorrected. Infra-red spectra were examined on ATRAlpha FTIR spectrophotometer (Billerica, MA, USA). ^1^H-NMR and ^13^C-NMR spectra examined on a Bruker AC-850 MHz apparatus (Bruker, Billerica, Massachusetts). Chemical shifts expressed as (ppm) relative to internal standard (TMS), and DMSO-d6 used as the solvent and in ^13^C-NMR the solvent was CDCl_3_ and DMSO mixture. CHN analyses and biological activity were achieved in Cairo University at Micro-Analytical Center. Spectral data of all compounds are available in Supplementary Materials.

#### 4.1.1. “*N*-(4-{[(7-methyl-8,10-dioxo-8,10-dihydroimidazo[2,1-b]pteridin-2-yl)methyl]amino}-benzoyl)glutamic acid” (**2**)

A mixture of folic acid (0.001 mol, 0.44 g) and ethyl pyruvate (0.001 mol, 0.11 g) dissolved in (15 mL) DMF and refluxed. After 8 hr (TLC, *R_F_* = 0.6, eluent: CH_2_Cl_2_) the solvent evaporated under vacuum, the semisolid product formed, poured onto ice, the solid resulted filtered off and crystallized from DMF-ethanol mixture (1:1) to give yellowish brown product. Yield, 87%, m.p. 223–225 °C. IR, 3437, 3407 cm^−1^ (2OH), 3239–3071 cm^−1^ (2NH), 2941 cm^−1^ (Ar-H), 2759 cm^−1^ (Aliphatic-H), 1689–1599 cm^−1^ (5C=O and C=N), 1495 cm^−1^ (C=C). ^1^H-NMR (DMSO *d_6_*, 850 MHz): δ = 1.30 (s, 3H, CH_3_), 1.89–2.01 (m, 2H, C*H_2_*CH_2_COOH), 2.72 (t, 2H, CH_2_C*H_2_*COOH), 4.25 (t, 1H, NHC*H*COOH), 4.46 (s, 2H, pteridine-C*H_2_*-N), 6.63 (d, 2H, N-Ph-(*H*)_ortho_), 7.24 (s, 1H, *H*N-Ph), 7.57 (d, 2H, N-Ph-(*H*)_meta_), 7.94 (s, 1H, N*H*CO), 8.63 (s,1H, pteridine-C_7_*H*), 11.48 (s,1H, CH_2_CH_2_COO*H*), 12.36 (s,1H, CH_2_COO*H*). ^13^C-NMR (DMSO *d_6_*, 200 MHz): δ = 24.51 (CH_3_), 30.89 (*C*H_2_CH_2_COOH), 33.21 (CH_2_*C*H_2_COOH), 45.95 (NH*C*H_2_), 50.15 (NH*C*H), 112.42 (N-Ph-(*C*)_ortho_), 113.45 (N-Ph-(*C*)_Para_), 121.76 (pteridine *C_4a_*), 128.00 (N-Ph-(*C*)_meta_), 147.28 (pteridine *C_6_*), 151.73 (pteridine *C_7_*), 152.77 (N-Ph-(*C*)), 154.88 (pteridine *C_8a_*), 157.47 (pteridine *C_2_*), 162.99 (pteridine *C_4_*), 167.65 NH*C*O, 174.25 (NHCH*C*OOH), 174.87 (CH_2_CH_2_*C*OOH). Anal. Calcd. for C_22_H_19_N_7_O_7_ (493.13): C, 53.55; H, 3.88; N, 19.87; found: C, 53.21; H, 3.62; N, 19.77.

#### 4.1.2. “*N*-[4-({[2-(dipyruvoylamino)-4-oxo-3,4-dihydropteridin-6-yl]methyl}-amino)benzoyl]-glutamic acid” (**3**)

A mixture of folic acid (0.001 mol, 0.44 g) and ethyl pyruvate (0.001 mol, 0.23 g) in DMF (20 mL) was refluxed for 2 h until the reaction completed (TLC, *R_F_* = 0.5, eluent: CH_2_Cl_2_). The solvent evaporated under vacuum. The precipitate formed, crystallized from EtOH to give yellow product. Yield, 52%, m.p. 188–190 °C. IR, 3541, 3408 cm^−1^ (2OH), 3225–3161 cm^−1^ (3NH), 3022–2941 cm^−1^ (Ar-H), 2791 cm^−1^ (Aliphatic-H), 1684–1599 cm^−1^ (8C=O and C=N), 1496 cm^−1^ (C=C). ^1^HNMR (DMSO *d_6_*, 850 MHz): δ = 1.89-2.01 (m, 2H, C*H_2_*CH_2_COOH), 2.21 (s, 6H, 2CH_3_), 2.72 (t, 2H, CH_2_C*H_2_*COOH), 4.25 (t, 1H, NHC*H*COOH), 4.46 (s, 2H, pteridine-C*H_2_*-N), 6.63 (d, 2H, N-Ph-(*H*)_ortho_), 7.24 (s, 1H, *H*N-Ph), 7.57 (d, 2H, N-Ph-(*H*)_meta_), 7.94 (s, 1H, N*H*CO), 8.63 (s,1H, pteridine-C_7_*H*), 10.15 (s, 1H, pteridine-N*H*), 11.45 (s,1H, CH_2_CH_2_COO*H*), 12.38 (s,1H, CH_2_COO*H*). ^13^C-NMR (DMSO *d_6_*, 200 MHz): δ = 26.79 (2CH_3_), 30.80 (*C*H_2_CH_2_COOH*)*, 34.28 (CH_2_*C*H_2_COOH), 45.97 (NH*C*H_2_), 52.35 (NH*C*H), 111.26 (N-Ph-(*C*)_ortho_), 112.56 (N-Ph-(*C*)_Para_), 121.61 (pteridine *C_4a_*), 128.85 (N-Ph-(*C*)_meta_), 148.38 (pteridine *C_6_*), 150.74 (pteridine *C_7_*), 151.73 (N-Ph-(*C*)), 154.28 (pteridine *C_8a_*), 156.28 (pteridine *C_2_*), 161.39 (N*C*OCOCH_3_, 162.35 (pteridine *C_4_*), 166.06 NH*C*O, 174.33 (NHCH*C*OOH), 174.41 (CH_2_CH_2_*C*OOH), 183.35 NCO*C*OCH_3_). Anal. Calcd. for C_25_H_23_N_7_O_10_ (581.49): C, 51.64; H, 3.99; N, 16.86; found: C, 51.51; H, 3.82; N, 16.77.

#### 4.1.3. “*N*-(4-{[(2-{[1-ethoxymethylene]amino}-4-oxo-3,4-dihydropteridin-6-yl)methyl]amino}-benzoyl)glutamic acid” (**4**)

Folic acid (0.01 mol, 4.4 g) added to triethyl orthoformate (8 mL, excess) and stirred under boiling for 6 h (TLC, *R_F_* = 0.8, eluent: CH_2_Cl_2_). The precipitate formed after cooling filtered and crystallized from EtOH to give yellow-brown powder. Yield, 85%, m.p. 220–222 °C. IR, 3470–3400 cm^−1^ (2OH), 3274–3115 cm^−1^ (3NH), 2974 cm^−1^ (Ar-H), 2803 cm^−1^ (Aliphatic-H), 1693–1601 cm^−1^ (4C=O and C=N). ^1^H-NMR (DMSO *d_6_*, 850 MHz): δ = 1.15 (t, 3H, CH_3_), 1.91–2.03 (m, 2H, C*H_2_*CH_2_COOH), 2.51 (t, 2H, CH_2_C*H_2_*COOH), 3.39 (q, 2H, CH_2_), 4.31 (t, 1H, NHC*H*COOH), 4.48 (s, 2H, pteridine-C*H_2_*-N), 6.64 (d, 2H, N-Ph-(*H*)_ortho_), 6.93 (s, 1H, *H*N-Ph), 7.64 (m, 2H, N-Ph-(*H*) _(meta)_), 7.65 (s, 1H, N=C*H*), 8.12 (s, 1H, N*H*CO), 8.79 (s,1H, pteridine-C_7_*H*), 10.38 (s, 1H, pteridine NH), 11.42 (s, 1H, CH_2_CH_2_COO*H*), 12.40 (s,1H, CH_2_COO*H*). Anal. Calcd. for C_22_H_23_N_7_O_7_ (497.46): C, 53.12; H, 4.66; N, 19.71; found: C, 52.89; H, 4.47; N, 19.54.

#### 4.1.4. “*N*-{4-[({2-[(ethoxycarbonyl)amino]-4-oxo-3,4-dihydropteridin-6-yl}methyl)amino]-benzoyl}glutamic acid” (**5**)

Folic acid (0.001 mol, 0.44 g), ethyl chloroformate (0.001 mol,0.11 g) and drops of TMA were mixed together in EtOH (12 mL) and refluxed for 1 h until the reaction completed (TLC, *R_F_* = 0.65, eluent: CH_2_Cl_2_). The solvent evaporated and the precipitate formed crystallized from EtOH to give yellow product. Yield, 87%, m.p. 218–220 °C. IR, 3438, 3422 cm^−1^ (2OH), 3251–3108 cm^−1^ (4NH), 3079–2939 cm^−1^ (Ar-H), 2782–2724 cm^−1^ (Aliphatic-H), 1690–1598 cm^−1^ (5C=O and C=N), 1496 cm^−1^ (C=C). ^1^H-NMR (DMSO *d_6_*, 850 MHz): δ = 1.14 (t, 3H, CH_2_C*H*_3_), 1.90–2.05 (m, 2H, C*H_2_*CH_2_COOH), 2.31 (t, 2H, CH_2_C*H_2_*COOH), 4.02 (q, 2H, C*H*_2_CH_3_), 4.08 (t, 1H, NHC*H*COOH), 4.53 (s, 2H, pteridine-C*H_2_*-N), 6.64 (d, 2H, N-Ph-(*H*)_ortho_), 7.64 (s, 1H, *H*N-Ph), 7.65 (d, 2H, N-Ph-(*H*)_meta_), 8.15 (s, 1H, N*H*CO), 8.21 (s,1H, pteridine-C_7_*H*), 8.72 (s, 1H, N*H*COOEt), 10.31 (s, 1H, pteridine NH), 11.51 (s,1H, CH_2_CH_2_COO*H*), 12.40 (s,1H, CH_2_COO*H*). ^13^C-NMR (DMSO *d_6_*, 200 MHz): δ = 25.09 (*C*H_3_CH_2_), 30.87 (*C*H_2_CH_2_COOH*)*, 34.11 (CH_2_*C*H_2_COOH), 41.20 (CH_3_*C*H_2_), 45.23 (NH*C*H_2_), 52.10 (NH*C*H), 111.44 (N-Ph-(*C*)_ortho_), 112.56 (N-Ph-(*C*)_Para_), 121.61 (pteridine *C_4a_*), 128.85 (N-Ph-(*C*)_meta_), 148.52 (pteridine *C_6_*), 150.70 (pteridine *C_7_*), 151.74 (N-Ph-(*C*)), 154.27 (pteridine *C_8a_*), 156.41 (pteridine *C_2_*), 161.89 (N*C*OOCH_2_CH_3_), 162.35 (pteridine *C_4_*), 166.06 (NH*C*O), 174.33 (NHCH*C*OOH), 174.41 (CH_2_CH_2_*C*OOH). Anal. Calcd. for C_22_H_23_N_7_O_8_ (513.46): C, 51.46; H, 4.51; N, 19.10; found: C, 51.25; H, 4.35; N, 18.84.

### 4.2. Synthesis of Derivatives ***6a**,**b*** (General Procedure)

Folic acid (0.001 mol, 0.4 g) and appropriate aldehyde (0.001 mol) mixed in glacial acetic acid (10 mL) and drops of hydrochloric acid (0.4 mL) were added, then, the mixture refluxed for 4 h (TLC, *R_F_* = 0.5–0.6, eluent: CH_2_Cl_2_). The formed precipitate filtered and crystallized from EtOH.

#### 4.2.1. “*N*-[4-({[2-(methyleneamino)-4-oxo-3,4-dihydropteridin-6-yl]methyl}-amino)benzoyl]glutamic acid” (**6a**)

Green powder. Yield, 81%, m.p. 210–212 °C. IR, 3418, 3403 cm^−1^ (2OH), 3284–3179 cm^−1^ (3NH), 2934 cm^−1^ (Ar-H), 2832 cm^−1^ (Aliphatic-H), 1690–1618 cm^−1^ (4C=O and C=N), 1590 cm^−1^ (C=C). ^1^H-NMR (DMSO *d_6_*, 850 MHz): δ = 1.88–2.07 (m, 2H, C*H_2_*CH_2_COOH), 2.71 (t, 2H, CH_2_C*H_2_*COOH), 4.27 (t, 1H, NHC*H*COOH), 4.48 (s, 2H, pteridine-C*H_2_*-N), 6.59 (d, 2H, N-Ph-(*H*)_ortho_), 7.23 (s, 1H, *H*N-Ph), 7.55 (m, 2H, N-Ph-(*H*) _(meta)_), 7.94 (s, 1H, N*H*CO), 7.99 (s, 2H, N=C*H*_2_), 8.64 (s,1H, pteridine-C_7_*H*), 10.33 (s, 1H, pteridine NH), 11.37 (s, 1H, CH_2_CH_2_COO*H*), 12.41 (s,1H, CH_2_COO*H*). ^13^C-NMR (DMSO *d_6_*, 200 MHz): δ = 28.15 (*C*H_2_CH_2_COOH*)*, 33.15 (CH_2_*C*H_2_COOH), 43.89 (NH*C*H_2_), 54.56 (NH*C*H), 112.25 (N-Ph-(*C*)_ortho_), 114.01 (N-Ph-(*C*)_Para_), 122.47 (pteridine *C_4a_*), 128.25 (N-Ph-(*C*)_meta_), 139.75 (N=C*H*_2_), 146.78 (pteridine *C_6_*), 151.20 (pteridine *C_7_*), 152.41 (N-Ph-(*C*)), 154.44 (pteridine *C_8a_*), 157.61 (pteridine *C_2_*), 163.36 (pteridine *C*O), 166.49 (NH*C*O), 175.21 (NHCH*C*OOH), 175.15 (CH_2_CH_2_*C*OOH). Anal. Calcd. for C_20_H_19_N_7_O_6_ (453.41): C, 52.98; H, 4.22; N, 21.62; found: C, 52.71; H, 4.01; N, 21.41.

#### 4.2.2. “*N*-[4-(((2-(Benzylideneamino)-4-oxo-3,4-dihydropteridin-6-yl)methyl)-amino)benzoyl)-glutamic acid” (**6b**)

Yellow powder. Yield, 62%, m.p. 224–226 °C. IR, 3412, 3399 cm^−1^ (2OH), 3279–3176 cm^−1^ (3NH), 2951 cm^−1^ (Ar-H), 2850 cm^−1^ (Aliphatic-H), 1687–1610 cm^−1^ (4C=O and C=N), 1550 cm^−1^ (C=C). ^1^H-NMR (DMSO *d_6_*, 850 MHz): δ = 1.85-2.04 (m, 2H, C*H_2_*CH_2_COOH), 2.73 (t, 2H, CH_2_C*H_2_*COOH), 4.22 (t, 1H, NHC*H*COOH), 4.45 (s, 2H, pteridine-C*H_2_*-N), 6.61 (d, 2H, N-Ph-(*H*)_ortho_), 7.22 (s, 1H, *H*N-Ph), 7.57 (m, 2H, N-Ph-(*H*) _meta_), 7.63 (d, 1H, benzylidene Ph-(*H*)_para_), 7.86 (d, 2H, benzylidene Ph-(*H*)_ortho_), 7.90 (m, 2H, benzylidene Ph-(*H*)_meta_), 7.93 (s, 1H, N*H*CO), 8.03 (s, 1H, benzylidene N=C-*H*), 8.60 (s,1H, pteridine-C_7_*H*), 10.24 (s, 1H, pteridine NH), 11.38 (s, 1H, CH_2_CH_2_COO*H*), 12.30 (s,1H, CH_2_COO*H*). ^13^C-NMR (DMSO *d_6_*, 200 MHz): δ = 30.75 (*C*H_2_CH_2_COOH), 33.10 (CH_2_*C*H_2_COOH), 45.94 (NH*C*H_2_), 50.07 (NH*C*H), 113.20 (N-Ph-(*C*)_ortho_), 114.23 (N-Ph-(*C*)_Para_), 121.36 (pteridine *C_4a_*), 128.22 (N-Ph-(*C*)_meta_), 128.09 (benzylidene Ph_(meta)_), 129.25 (benzylidene Ph_(ortho)_), 131.70 (benzylidene Ph_(para)_), 133.01 (benzylidene CH-*Ph*), 146.20 (pteridine *C_6_*), 151.52 (pteridine *C_7_*), 152.44 (N-Ph-(*C*)), 154.40 (pteridine *C_8a_*), 157.13 (pteridine *C_2_*), 163.36 (pteridine *C*O), 164.21 (benzylidene N=C-*H*), 167.89 NH*C*O, 174.25 (NHCH*C*OOH), 174.54 (CH_2_CH_2_*C*OOH). Anal. Calcd. for C_26_H_23_N_7_O_6_ (529.50): C, 58.98; H, 4.38; N, 18.52; found: C, 58.74; H, 4.22; N, 18.46.

#### 4.2.3. “*N*-{4-[({2-[(hydrazinocarbonothioyl)amino]-4-oxo-3,4-dihydropteridin-6-yl}methyl)-amino]benzoyl}glutamic acid” (**7**)

Folic acid (0.001 mol, 0.44 g) dissolved with thioformic acid hydrazide (0.001 mol, 0.1 g) in EtOH/DMF (2:1–15 mL) and stirred under reflux for 10 h. (TLC, *R_F_* = 0.4, eluent: CH_2_Cl_2_). The precipitate formed filtered and crystallized from EtOH to give yellow powder. Yield 91%, m.p. over 300 °C. IR, 3450–3342 cm^−1^ (2OH), 3342–3049 cm^−1^ (NH_2_ and 5NH), 2940 cm^−1^ (Ar-H), 2875 cm^−1^ (Aliphatic-H), 1685–1600 cm^−1^ (4C=O and C=N), 1338 cm^−1^ (C=S). ^1^H-NMR (DMSO *d_6_*, 850 MHz): δ = 1.89–2.01 (m, 2H, C*H_2_*CH_2_COOH), 2.49 (t, 2H, CH_2_C*H_2_*COOH), 4.09 (t, 1H, NHC*H*COOH), 4.44 (s, 2H, pteridine-C*H_2_*-N), 5.69 (s, 2H, NH_2_), 6.91(d, 2H, N-Ph-(*H*)_ortho_), 6.99 (s, 1H, *H*N-Ph), 7.60 (m, 6H, N-Ph-(*H*) _(meta)_), 8.10 (s, 1H, N*H*CO), 8.63 (s,1H, pteridine-C_7_*H*), 10.31 (s, 1H, pteridine NH), 11.31 (s, 1H, N*H*NH_2_), 11.32 (s, 1H, CH_2_CH_2_COO*H*), 12.18 (s,1H, CH_2_COO*H*), 12.66 (s, 1H, N*H*-pyrimidine), 12.89 (s, 1H, N*H*-C=S). Calcd. for C_20_H_21_N_9_O_6_S (515.50): C, 46.60; H, 4.11; N, 24.45; found: C, 46.43; H, 4.01; N, 24.18.

#### 4.2.4. “N,N’-bis[6-({[4-(N-glutamincarbonyl)phenyl]amino}-methyl)-4-oxo-3,4-dihydropteridin-2-yl]decanediamide” (**8**)

Compound **1** (0.001 mol, 0.88 g) and sebacoyl chloride (0.001 mol, 0.24 g) and drops of TMA in DMF (10 mL) was refluxed for 6 h (TLC, *R_F_* = 0.6, eluent: CH_2_Cl_2_). The mixture was poured into ice-cold water, the precipitate obtained crystallized from EtOH to give orange crystals. Yield, 72%, m.p. 184–186 °C. IR, 3432, 3415 cm^−1^ (2OH), 3270–3160 cm^−1^ (4NH), 2951 cm^−1^ (Ar-H), 2850 cm^−1^ (Aliphatic-H), 1688–1621 cm^−1^ (5C=O and C=N), 1347 cm^−1^ (C=S). ^1^H-NMR (DMSO *d_6_*, 850 MHz): δ = 1.03 (m, 4H, 2CH_2_, CH_2_C*H*_2_CH_2_CH_2_CONH), 1.26 (m, 4H, 2CH_2_, C*H*_2_CH_2_CH_2_CH_2_CONH), 1.90–2.04 (t, 2H, C*H_2_*CH_2_COOH), 2.23 (m, 4H, 2CH_2_, CH_2_CH_2_C*H*_2_CH_2_CONH), 2.71 (t, 2H, CH_2_C*H_2_*COOH), 3.34 (t, 4H, 2CH_2_, CH_2_CH_2_CH_2_C*H*_2_CONH), 4.24 (t, 1H, NHC*H*COOH), 4.51 (s, 2H, pteridine-C*H_2_*-N), 6.61 (d, 2H, N-Ph-(*H*)_ortho_), 7.21 (s, 1H, *H*N-Ph), 7.49 (m, 2H, N-Ph-(*H*) _(meta)_), 7.90 (s, 1H, N*H*CO), 8.61 (s,1H, pteridine-C_7_*H*), 10.37 (s, 1H, pteridine NH), 11.12 (s, 2H, 2NH, CH_2_CH_2_CH_2_CH_2_CON*H*), 11.43 (s, 1H, CH_2_CH_2_COO*H*), 12.34 (s,1H, CH_2_COO*H*). Anal. Calcd. for C_48_H_52_N_14_O_14_ (1049.01): C, 54.96; H, 5.00; N, 18.69; found: C, 54.78; H, 4.81; N, 18.52.

#### 4.2.5. “*N*-[4-({[4-oxo-2-(3-oxo-5-thioxo-1,2,4-triazolidin-4-yl)-3,4-dihydro-pteridin-6-yl]methyl}amino)benzoyl]glutamic acid” (**9**)

Compound **7** (0.001 mol, 0.51 g) was refluxed for 16 h with ethyl chloroformate (0.001 mol, 0.11 g) and TMA (3 drops) in EtOH (15 mL) (TLC, *R_F_* = 0.8, eluent: CH_2_Cl_2_). A yellow powder formed which filtered and crystallized from EtOH. Yield, 78%, m.p. over 300 °C. IR, 3412–3378 cm^−1^ (2OH), 3331–3149 cm^−1^ (5NH), 2961 cm^−1^ (Ar-H), 2867 cm^−1^ (Aliphatic-H), 1681–1618 cm^−1^ (5C=O and C=N), 1350 cm^−1^ (C=S). ^1^H-NMR (DMSO *d_6_*, 850 MHz): δ = 1.90–2.03 (m, 2H, C*H_2_*CH_2_COOH), 2.497 (t, 2H, CH_2_C*H_2_*COOH), 4.04 (N-C*H*COOH), 4.48 (s, 2H, pteridine-C*H_2_*-N), 6.931(d, 2H, N-Ph-(*H*)_ortho_), 6.95 (s, 1H, *H*N-Ph), 7.63 (m, 6H, N-Ph-(*H*) _(meta)_), 8.12 (s, 1H, N*H*CO), 8.63 (s,1H, pteridine-C_7_*H*), 10.13 (s, 1H, triazolidine N*H*C=S), 10.37 (s, 1H, pteridine NH), 11.31 (s, 1H, CH_2_CH_2_COO*H*), 11.60 (s, 1H, triazolidine N*H*C=O), 12.21 (s,1H, CH_2_COO*H*), 13.01 (s, 1H, N*H*-pyrimidine). ^13^C-NMR (DMSO *d_6_*, 200 MHz): δ = 29.66 (*C*H_2_CH_2_COOH), 31.23 (CH_2_*C*H_2_COOH), 45.18 (NH*C*H_2_), 53.41 (NH*C*H), 112.25 (N-Ph-(*C*)_ortho_), 114.01 (N-Ph-(*C*)_Para_), 122.44 (pteridine *C_4a_*), 127.90 (N-Ph-(*C*)_meta_), 148.02 (pteridine *C_6_*), 151.48 (pteridine *C_7_*), 153.14 (N-Ph-(*C*)), 154.22 (pteridine *C_8a_*), 156.32 (triazolidine C=O), 161.01 (pteridine *C_2_*), 165.90 (pteridine *C*O), 166.05 (NH*C*O), 174.45 (NHCH*C*OOH), 174.58 (CH_2_CH_2_*C*OOH), 182.37 (triazolidine C=S). Calcd. for C_21_H_19_N_9_O_7_S (541.50): C, 46.58; H, 3.54; N, 23.28; found: C, 46.33; H, 3.40; N, 23.12.

#### 4.2.6. “*N*-(4-{[(9-amino-8-cyano-11-oxo-7-phenyl-11H-pyrimido[2,1-b]pteridin-2-yl)methyl]-amino}benzoyl)glutamic acid” (**10**)

A mixture of folic acid (0.001 mol, 0.44 g), malononitrile (0.001 mol, 0.66 g), benzaldehyde (0.11 g, 1 mmol), and drops of TMA in ethanol (15 mL) was stirred under reflux for 4 h (TLC, *R_F_* = 0.4, eluent: CH_2_Cl_2_). The reaction cooled at rt (room temperature). then, the precipitate formed filtered and crystallized from EtOH to give orange crystals. Yield 80%, m.p. 228–230 °C. IR, 3420–3400 cm^−1^ (2OH), 3250–3071 cm^−1^ (NH_2_ and 2NH), 2950 cm^−1^ (Ar-H), 2850 cm^−1^ (Aliphatic-H), 2217 cm^−1^ (CN), 1682–1598 cm^−1^ (4C=O and C=N). ^1^H-NMR (DMSO *d_6_*, 850 MHz): δ = 1.89–2.02 (m, 2H, C*H_2_*CH_2_COOH), 2.50 (t, 2H, CH_2_C*H_2_*COOH), 4.02 (t, 1H, NHC*H*COOH), 4.48 (s, 2H, pteridine-C*H_2_*-N), 6.65 (s, 2H, NH_2_), 6.93 (d, 2H, N-Ph-(*H*)_ortho_), 6.95 (s, 1H, *H*N-Ph), 7.62–7.96 (m, 7H, N-Ph-(*H*) _(meta)_ and pyrimidine-4-*Ph*), 8.12 (s, 1H, N*H*CO), 8.65 (s,1H, pteridine-C_7_*H*), 11.45 (s, 1H, CH_2_CH_2_COO*H*), 12.18 (s,1H, CH_2_COO*H*). Calcd. for C_29_H_23_N_9_O_6_ (593.55): C, 58.68; H, 3.91; N, 21.24; found: C, 58.42; H, 3.68; N, 21.01.

#### 4.2.7. “*N*-(4-{[(7,9-dimethyl-11-oxo-11H-pyrimido[2,1-b]pteridin-2-yl)methyl]-amino}benzoyl) glutamic acid” (**11**)

Folic acid (0.001 mol, 0.44 g) added to acetylacetone (0.001 mol, 0.1 g) in DMF (12 mL) and stirred under reflux for 4 h (TLC, *R_F_* = 0.8, eluent: CH_2_Cl_2_). The precipitate formed crystallized from EtOH to give reddish-brown product. Yield, 93%, m.p. 252–254 °C. IR, 3421–3403 cm^−1^ (2OH), 3266–3214 cm^−1^ (2NH), 2952 cm^−1^ (Ar-H), 2853 cm^−1^ (Aliphatic-H), 1681–1621 cm^−1^ (4C=O and C=N). ^1^H-NMR (DMSO *d_6_*, 850 MHz): δ = 1.91–2.03 (m, 2H, C*H_2_*CH_2_COOH), 2.26 (s, 3H, CH_3_), 2.40 (s, 3H, CH_3_), 2.53 (t, 2H, CH_2_C*H_2_*COOH), 4.24 (t, 1H, NHC*H*COOH), 4.46 (s, 2H, pteridine-C*H_2_*-N), 6.53 (d, 2H, N-Ph-(*H*)_ortho_), 6.90 (s, 1H, *H*N-Ph), 7.27 (s, 1H, pyrimidine C*H*), 7.56 (m, 2H, N-Ph-(*H*) _(meta)_), 8.01 (s, 1H, N*H*CO), 8.62 (s,1H, pteridine-C_7_*H*), 11.27 (s, 1H, CH_2_CH_2_COO*H*), 12.06 (s,1H, CH_2_COO*H*). ^13^CNMR (DMSO *d_6_*, 200 MHz): δ = 24.30 (CH_3_), 26.45 (CH_3_), 29.28 (*C*H_2_CH_2_COOH*)*, 31.11 (CH_2_*C*H_2_COOH), 45.04 (NH*C*H_2_), 48.47 (pyrimidine CH_2_), 52.49 (NH*C*H), 111.27 (N-Ph-(*C*)_ortho_), 112.58 (N-Ph-(*C*)_Para_), 120.95 (pyrimidine *C_5_*), 121.68 (pteridine *C_4a_*), 127.98 (N-Ph-(*C*)_meta_), 148.36 (pteridine *C_6_*), 150.72 (pteridine *C_7_*), 151.71 (N-Ph-(*C*)), 154.35 (pteridine *C_8a_*), 161.44 (pteridine *C_2_*), 165.95 (pteridine *C*O), 166.05 (NH*C*O), 171.79 (pyrimidine N=CH), 174.45 (NHCH*C*OOH), 174.58 (CH_2_CH_2_*C*OOH). Anal. Calcd. for C_24_H_23_N_7_O_6_ (505.48): C, 57.03; H, 4.59; N, 19.40; found: C, 56.71; H, 4.26; N, 19.11.

#### 4.2.8. “*N*-(4-{[(13a-hydroxy-5,12-dioxo-12,13a-dihydro-5H-indeno-[2′,1′:4,5]imidazo[2,1-b]pteridin-10-yl)methyl]amino}benzoyl)-glutamic acid” (**12**)

Folic acid (0.001 mol, 0.44 g) and ninhydrin (0.001 mol, 0.18 g) in EtOH (12 mL) were refluxed for 4 h (TLC, *R_F_* = 0.75, eluent: CH_2_Cl_2_). A yellowish orange crystal formed on hot which, filtered and washed with EtOH. Yield 74%, m.p. 260–262 °C. IR, 3448–3343 cm^−1^ (3OH), 3248–3073 cm^−1^ (2NH), 2938 cm^−1^ (Ar-H), 2788 cm^−1^ (Aliphatic-H), 1682–1599 cm^−1^ (5C=O and C=N). ^1^H-NMR (DMSO *d_6_*, 850 MHz): δ = 1.94–2.04 (m, 2H, C*H_2_*CH_2_COOH), 2.51 (t, 2H, CH_2_C*H_2_*COOH), 4.04 (t, 1H, NHC*H*COOH), 4.47 (s, 2H, pteridine-C*H_2_*-N), 5.36 (s, 1H, imidazoloindene OH), 6.91(d, 2H, N-Ph-(*H*)_ortho_), 6.95 (s, 1H, *H*N-Ph), 7.62-7.96 (m, 6H, N-Ph-(*H*) _(meta)_ and indene-*Ph*), 8.11 (s, 1H, N*H*CO), 8.63 (s,1H, pteridine-C_7_*H*), 11.37 (s, 1H, CH_2_CH_2_COO*H*), 12.21 (s,1H, CH_2_COO*H*). Calcd. for C_28_H_21_N_7_O_8_ (583.51): C, 57.63; H, 3.63; N, 16.80; found: C, 57.47; H, 3.41; N, 16.53.

#### 4.2.9. “*N*-(4-{[(7-methyl-9,11-dioxo-6,11-dihydro-9H-pyrimido[2,1-b]pteridin-2-yl)methyl]-amino}benzoyl)glutamic acid” (**13**)

Folic acid (0.001 mol, 0.44 g) and ethyl acetoacetate (0.001 mol, 0.13 g) in DMF (13 mL) stirred under reflux for 5 h (TLC, *R_F_* = 0.75, eluent: CH_2_Cl_2_). The precipitate separated after cooling crystallized from ethanol to give orange crystals. Yield, 81%, m.p. 246–248 °C. IR, 3418–3412 cm^−1^ (2OH), 3271–3223 cm^−1^ (3NH), 2956 cm^−1^ (Ar-H), 2850 cm^−1^ (Aliphatic-H), 1677–1620 cm^−1^ (5C=O and C=N). ^1^H-NMR (DMSO *d_6_*, 850 MHz): δ = 1.90–2.00 (m, 2H, C*H_2_*CH_2_COOH), 2.25 (s, 3H, CH_3_), 2.55 (t, 2H, CH_2_C*H_2_*COOH), 4.20 (t, 1H, N-C*H*COOH), 4.43 (s, 2H, pteridine-C*H_2_*-N), 6.51 (d, 2H, N-Ph-(*H*)_ortho_), 6.93 (s, 1H, *H*N-Ph), 7.21 (s, 1H, pyrimidine C*H*), 7.58 (m, 2H, N-Ph-(*H*) _(meta)_), 8.04 (s, 1H, N*H*CO), 8.66 (s,1H, pteridine-C_7_*H*), 10.95 (s, 1H, pyrimidine N*H*), 11.24 (s, 1H, CH_2_CH_2_COO*H*), 12.14 (s,1H, CH_2_COO*H*). Anal. Calcd. for C_23_H_21_N_7_O_7_ (507.46): C, 54.44; H, 4.17; N, 19.32; found: C, 54.13; H, 4.08; N, 19.01.

#### 4.2.10. “*N*-(4-{[(7-amino-9,11-dioxo-8,11-dihydro-9H-pyrimido[2,1-b]pteridin-2-yl)methyl]-amino}benzoyl)glutamic acid” (**14**)

Folic acid (0.001 mol, 0.44 g) and ethyl cyanoacetate (0.001 mol, 0.13 g) in DMF (15 mL) stirred at boiling point for 4 h until the reaction finished (TLC, *R_F_* = 0.7, eluent: CH_2_Cl_2_). The product obtained after solvent evaporation crystallized from EtOH-DMF to give reddish brown product. Yield, 79%, m.p. 231–2330 °C. IR, 3424, 3412 cm^−1^ (2OH), 3278–3189 cm^−1^ (NH_2_ and 2NH), 2911 cm^−1^ (Ar-H), 2843 cm^−1^ (Aliphatic-H), 1679–1624 cm^−1^ (5C=O and C=N), 1592 cm^−1^ (C=C). ^1^H-NMR (DMSO *d_6_*, 850 MHz): δ = 1.74–2.10 (m, 2H, C*H_2_*CH_2_COOH), 2.73 (t, 2H, CH_2_C*H_2_*COOH), 2.89 (s, 2H, pyrimidine CH_2_), 4.22 (t, 1H, NHC*H*COOH), 4.51 (s, 2H, pteridine-C*H_2_*-N), 6.63 (d, 2H, N-Ph-(*H*) _ortho_), 7.23 (s, 1H, *H*N-Ph), 7.56 (m, 2H, N-Ph-(*H*)_(meta)_), 7.98 (s, 1H, N*H*CO), 8.06 (s, 2H, N*H*_2_), 8.61 (s,1H, pteridine-C_7_*H*), 11.46 (s, 1H, CH_2_CH_2_COO*H*), 12.47 (s,1H, CH_2_COO*H*). ^13^C-NMR (DMSO *d_6_*, 200 MHz): δ = 28.10 (*C*H_2_CH_2_COOH*)*, 33.19 (CH_2_*C*H_2_COOH), 43.44 (NH*C*H_2_), 43.05 (pyrimidine CH_2_), 54.50 (NH*C*H), 113.22 (N-Ph-(*C*)_ortho_), 114.61 (N-Ph-(*C*)_Para_), 123.34 (pteridine *C_4a_*), 128.24 (N-Ph-(*C*)_meta_), 146.32 (pteridine *C_6_*), 151.22 (pteridine *C_7_*), 152.11 (N-Ph-(*C*)), 154.79 (pteridine *C_8a_*), 160.09 (pteridine *C_2_*), 165.36 (pteridine *C*O), 166.22 (NH*C*O), 171.03 (pyrimidine N=CH), 174.12 (NHCH*C*OOH), 175.00 (CH_2_CH_2_*C*OOH). Anal. Calcd. for C_22_H_20_N_8_O_7_ (508.44): C, 51.97; H, 3.96; N, 22.04; found: C, 51.79; H, 3.81; N, 21.91.

#### 4.2.11. “*N*-(4-{[(7,10-dioxo-6,7,8,10-tetrahydroimidazo[2,1-b]pteridin-2-yl)methyl]-amino}-benzoyl)glutamic acid” (**15**)

Ethyl chloroacetate (0.001 mol, 0.12 g) and folic acid (0.001 mol, 0.44 g) in DMF (11 mL) stirred under reflux for 4.5 h (TLC, *R_F_* = 0.8, eluent: CH_2_Cl_2_). The precipitate separated after cooling crystallized from EtOH to yield orange crystals. Yield, 88%, m.p. 221–224 °C. IR, 3423–3401 cm^−1^ (2OH), 3251–3204 cm^−1^ (3NH), 2927 cm^−1^ (Ar-H), 2854 cm^−1^ (Aliphatic-H), 1668–1618 cm^−1^ (5C=O and C=N). ^1^H-NMR (DMSO *d_6_*, 850 MHz): δ = 1.93–2.04 (m, 2H, C*H_2_*CH_2_COOH), 2.55 (t, 2H, CH_2_C*H_2_*COOH), 3.51 (s, 2H, imidazolidinone C*H*_2_), 4.26 (t, 1H, NHC*H*COOH), 4.48 (s, 2H, pteridine-C*H_2_*-N), 6.53 (d, 2H, N-Ph-(*H*)_ortho_), 6.92 (s, 1H, *H*N-Ph), 7.57 (m, 2H, N-Ph-(*H*) _(meta)_), 8.03 (s, 1H, N*H*CO), 8.64 (s,1H, pteridine-C_7_*H*), 10.87 (s, 1H, imidazole N*H*), 11.28 (s, 1H, CH_2_CH_2_COO*H*), 12.11 (s,1H, CH_2_COO*H*). Anal. Calcd. for C_21_H_19_N_7_O_7_ (481.42): C, 52.39; H, 3.98; N, 20.37; found: C, 52.00; H, 3.71; N, 20.19.

#### 4.2.12. “*N*-{4-[({2-[({2-[amino(imino)methyl]hydrazino}-carbonyl)amino]-4-oxo-3,4-dihydropteridin-6-yl}methyl)amino]benzoyl}-glutamic acid” (**16**)

Compound **15** (0.001 mol, 0.51 g) and aminoguanidinium hydrocarbonate (0.001 mol, 0.14 g) in glacial acetic acid (15 mL) was stirred under reflux for 3 h (TLC, *R_F_* = 0.6, eluent: CH_2_Cl_2_). A brownish powder formed on hot, the precipitate filtered while hot and washed with ethanol. Yield 87%, m.p. 274–276 °C. IR, 3439–3410 cm^−1^ (2OH), 3319–3161 cm^−1^ (NH_2_ and 7 NH), 2954 cm^−1^ (Ar-H), 2851 cm^−1^ (Aliphatic-H), 1678–1620 cm^−1^ (5C=O and C=N). ^1^H-NMR (DMSO *d_6_*, 850 MHz): δ = 1.93–2.05 (m, 2H, C*H_2_*CH_2_COOH), 2.55 (t, 2H, CH_2_C*H_2_*COOH), 4.31 (t, 1H, NHC*H*COOH), 4.54 (s, 2H, pteridine-C*H_2_*-N), 6.27 (s, 2H, NH_2_), 6.63 (d, 2H, N-Ph-(*H*)_ortho_), 6.91 (s, 1H, *H*N-Ph), 7.65 (m, 2H, N-Ph-(*H*) _(meta)_), 7.80 (s, 1H, C=NH), 8.14 (s, 1H, N*H*CO), 8.51 (s, 1H, pteridine-N*H*CONH), 8.82 (s,1H, pteridine-C_7_*H*), 10.17 (s, 1H, N*H*-NH), 10.31 (s, 1H, pteridine N*H*), 10.99 (s, 1H, NH-N*H*), 11.40 (s, 1H, CH_2_CH_2_COO*H*), 12.30 (s,1H, CH_2_COO*H*). Anal. Calcd. for C_21_H_23_N_11_O_7_ (541.48): C, 46.58; H, 4.28; N, 28.45; found: C, 46.36; H, 4.09; N, 28.31.

#### 4.2.13. “*N*-{4-[({2-[(hydrazinocarbonyl)amino]-4-oxo-3,4-dihydropteridin-6-yl}methyl)amino]-benzoyl}glutamic acid” (**17**)

Compound **15** (0.51g, 1 mmol) and NH_2_NH_2_ (excess, 3 mL) in DMF (12 mL) were refluxed for 5 h (TLC, *R_F_* = 0.5, eluent: CH_2_Cl_2_). The solution concentrated under vacuum and poured onto crushed ice, an orange compound resulted, crystallized from DMF:EtOH mixture 1:3 to give yellowish powder. Yield, 65%, m.p. 231–233 °C. IR, 3411, 3386 cm^−1^ (2OH), 3309–3214 cm^−1^ (NH_2_ and 5NH), 3001 cm^−1^ (Ar-H), 2876 cm^−1^ (Aliphatic-H), 1686–1618 cm^−1^ (5C=O and C=N). ^1^H-NMR (DMSO *d_6_*, 850 MHz): δ = 1.93–2.02 (m, 2H, C*H_2_*CH_2_COOH), 2.42 (t, 2H, CH_2_C*H_2_*COOH), 4.09 (t, 1H, NHC*H*COOH), 4.33 (s, 2H, s, 1H, NHCONHN*H_2_*_),_ 4.53 (s, 2H, pteridine-C*H_2_*-N), 6.61 (d, 2H, N-Ph-(*H*)_ortho_), 7.62 (s, 1H, *H*N-Ph), 7.68 (d, 2H, N-Ph-(*H*)_meta_), 8.11 (s, 1H, N*H*CO), 8.22 (s,1H, pteridine-C_7_*H*), 8.69 (s, 1H, NHCON*H*NH_2_), 9.61 (s, 1H, N*H*CONHNH_2_), 10.34 (s, 1H, pteridine NH), 11.41 (s,1H, CH_2_CH_2_COO*H*), 12.34 (s,1H, CH_2_COO*H*). ^13^C-NMR (DMSO *d_6_*, 200 MHz): δ = 31.18 (*C*H_2_CH_2_COOH), 35.41 (CH_2_*C*H_2_COOH), 47.13 (NH*C*H_2_), 51.99 (NH*C*H), 112.12 (N-Ph-(*C*)_ortho_), 114.43 (N-Ph-(*C*)_Para_), 121.67 (pteridine *C_4a_*), 129.11 (N-Ph-(*C*)_meta_), 147.71 (pteridine *C_6_*), 151.58 (pteridine *C_7_*), 151.78 (N-Ph-(*C*)), 155.41 (pteridine *C_8a_*), 158.18 (pteridine *C_2_*), 161.89 (N*C*ONHNH_2_), 162.11 (pteridine *C_4_*), 166.43 (NH*C*O), 175.81 (NHCH*C*OOH), 177.71 (CH_2_CH_2_*C*OOH). Anal. Calcd. for C_20_H_21_N_9_O_7_ (499.44): C, 48.10; H, 4.24; N, 25.24; found: C, 47.72; H, 4.11; N, 25.18.

#### 4.2.14. “*N*-[4-({[2-({[(carboxymethyl)amino]carbonyl}amino)-4-oxo-3,4-dihydropteridin-6-yl]methyl}amino)benzoyl]glutamic acid” (**18**)

Compound **15** (0.001 mol, 0.5 g) and glycine (0.001 mol, 0.08 g) in DMF/ H_2_O mixture (9:1, 10 mL) was refluxed for 4 h (TLC, *R_F_* = 0.70, eluent: CH_2_Cl_2_). The solvent concentrated by evaporation. The brown solid formed after pouring on crushed ice crystallized from EtOH. Yield, 83%, m.p. 210–212 °C. IR, 3451–3410 cm^−1^ (3OH), 3233–3185 cm^−1^ (3NH), 2971 cm^−1^ (Ar-H), 2857 cm^−1^ (Aliphatic-H), 1674–1623 cm^−1^ (6C=O and C=N). ^1^H-NMR (DMSO *d_6_*, 850 MHz): δ = 1.92–2.01 (m, 2H, C*H_2_*CH_2_COOH), 2.55 (t, 2H, CH_2_C*H_2_*COOH), 4.03 (s, 2H, NHC*H_2_*COOH), 4.33 (t, 1H, NHC*H*COOH), 4.51 (s, 2H, pteridine-C*H_2_*-N), 6.58 (d, 2H, N-Ph-(*H*)_ortho_), 6.96 (s, 1H, *H*N-Ph), 7.61 (m, 2H, N-Ph-(*H*) _(meta)_), 8.01 (s, 1H, N*H*CO), 8.66 (s,1H, pteridine-C_7_*H*), 8.78 (s, 1H, N*H*CH_2_COOH), 10.31 (s, 1H, pteridine N*H*), 11.31 (s, 1H, CH_2_CH_2_COO*H*), 11.60 (s,1H, CH_2_COO*H*), 12.22 (s, 1H, NHCH_2_COO*H*). ^13^CNMR (DMSO *d_6_*, 200 MHz): δ = 25.08 (CH_3_), 30.19 (*C*H_2_CH_2_COOH), 34.13 (CH_2_*C*H_2_COOH), 41.10 (glycine CH_2_), 45.90 (NH*C*H_2_), 51.21 (NH*C*H), 111.18 (N-Ph-(*C*)_ortho_), 120.99 (N-Ph-(*C*)_Para_), 127.97 (pteridine *C_4a_*), 128.69 (N-Ph-(*C*)_meta_), 148.09 (pteridine *C_6_*), 150.67 (pteridine *C_7_*), 150.91 (N-Ph-(*C*)), 154.69 (pteridine *C_8a_*), 156.30 (pteridine *C_2_*), 160.91 (pteridine *C_4_*), 161.53 (NHCONH), 166.52 NH*C*O, 172.09 (NHCH*C*OOH), 173.17 (glycine CO), 174.51 (CH_2_CH_2_*C*OOH). Anal. Calcd. for C_22_H_22_N_8_O_9_ (542.46): C, 48.71; H, 4.09; N, 20.66; found: C, 48.53; H, 3.78; N, 20.51.

#### 4.2.15. “*N*-[4-({[2-({[2-(mercaptocarbonothioyl)hydrazino]-carbonyl}amino)-4-oxo-3,4-dihydropteridin-6-yl]methyl}amino)benzoyl]glutamic acid” (**19**)

Compound **15** (0.001 mol, 0.51 g) and thioformic acid hydrazide (0.001 mol, 0.1 g) in DMSO (12 mL) were refluxed for 5 h (TLC, *R_F_* = 0.4, eluent: CH_2_Cl_2_). The product formed after pouring onto crushed ice crystallized from DMF/EtOH 1:1 to yield brown powder. Yield, 84%, m.p. 281–283 °C. IR, 3423–3402 cm^−1^ (2OH), 3251–3147 cm^−1^ (6NH), 2928 cm^−1^ (Ar-H), 2850 cm^−1^ (Aliphatic-H), 2627 cm^−1^ (SH), 1678–1621 cm^−1^ (5C=O and C=N), 1351 cm^−1^ (C=S). ^1^H-NMR (DMSO *d_6_*, 850 MHz): δ = 1.89–2.04 (m, 2H, C*H_2_*CH_2_COOH), 2.50 (t, 2H, CH_2_C*H_2_*COOH), 4.08 (s, 2H, NHC*H_2_*COOH), 4.31 (t, 1H, NHC*H*COOH), 4.54 (s, 2H, pteridine-C*H_2_*-N), 6.54 (d, 2H, N-Ph-(*H*)_ortho_), 6.96 (s, 1H, *H*N-Ph), 7.62 (m, 2H, N-Ph-(*H*) _(meta)_), 8.09 (s, 1H, N*H*CO), 8.61 (s,1H, pteridine-C_7_*H*), 10.36 (s, 1H, pteridine N*H*), 11.17 (s, 1H, N*H*-NH), 11.38 (s, 1H, NH-N*H*), 11.43 (s, 1H, CH_2_CH_2_COO*H*), 12.27 (s,1H, CH_2_COO*H*), 13.98 (s, 1H, S*H*). Anal. Calcd. for C_21_H_21_N_9_O_7_S_2_ (575.58): C, 43.82; H, 3.68; N, 21.90; S, 11.14; found: C, 43.57; H, 3.49; N, 21.77; S, 10.91.

### 4.3. Reaction of Compound 15 with Sulfa Drugs (***20a**,**b***): General Procedure

A mixture of **15** (0.001 mol, 0.51 g) and appropriate sulfa drug (0.001 mol) in DMF (15 mL) was stirred under reflux for 4h (TLC, *R_F_* = 0.45, eluent: CH_2_Cl_2_). The precipitate formed crystallized from EtOH to produce yellow to orange powder.

#### 4.3.1. “*N*-(4-{[(4-oxo-2-{[({4-[(pyrimidin-2-ylamino)sulfonyl]-phenyl}amino)carbonyl]-amino}-3,4-dihydropteridin-6-yl)methyl]amino}-benzoyl)glutamic acid” (**20a**)

Yellowish orange, yield, 77%, m.p. 238–240 °C. IR, 3438–3417 cm^−1^ (2OH), 3331–3199 cm^−1^ (6NH), 2952 cm^−1^ (Ar-H), 2853 cm^−1^ (Aliphatic-H), 1684–1623 cm^−1^ (5C=O and C=N). ^1^H-NMR (DMSO *d_6_*, 850 MHz): δ = 1.88–2.04 (m, 2H, C*H_2_*CH_2_COOH), 2.51 (t, 2H, CH_2_C*H_2_*COOH), 4.34 (t, 1H, NHC*H*COOH), 4.55 (s, 2H, pteridine-C*H_2_*-N), 6.42 (d, 2H, *J* = 8.4 Hz, benzene C_2_*H*,C_6_*H*), 6.65 (d, 2H, N-Ph-(*H*)_ortho_), 6.81 (d, 2H, *J* = 8.4 Hz, benzene C_3_*H*,C_5_*H*), 6.92 (s, 1H, *H*N-Ph), 7.66 (m, 2H, N-Ph-(*H*) _(meta)_), 8.17 (s, 1H, N*H*CO), 8.41 (t, 1H, pyrimidine C_3_*H*), 8.53 (s, 1H, pteridine-N*H*CONH), 8.64 (d, 2H, pyrimidine C_2_*H*,C_4_*H*), 8.77 (s,1H, pteridine-C_7_*H*), 8.99 (s, 1H, pteridine-NHCON*H*), 10.31 (s, 1H, pteridine N*H*), 11.38 (s, 1H, CH_2_CH_2_COO*H*), 12.27 (s,1H, CH_2_COO*H*), 12.83 (s, 1H, SO_2_N*H*). Anal. Calcd. for C_30_H_27_N_11_O_9_S (717.67): C, 50.21; H, 3.79; N, 21.47; S, 4.47; found: C, 49.91; H, 3.58; N, 21.62; S, 4.30.

#### 4.3.2. “*N*-[4-({[2-({[(4-{[(4,6-dimethylpyrimidin-2-yl)amino]-sulfonyl}phenyl)amino]-carbonyl}amino)-4-oxo-3,4-dihydropteridin-6-yl]methyl}amino) benzoyl]glutamic acid” (**20b**)

Yield, 72%, Orange powder, m.p. 246–248 °C. IR, 3442–3421 cm^−1^ (2OH), 3325–3212 cm^−1^ (6NH), 2975 cm^−1^ (Ar-H), 2857 cm^−1^ (Aliphatic-H), 1680–1621 cm^−1^ (5C=O and C=N). ^1^H-NMR (DMSO *d_6_*, 850 MHz): δ = 1.91–2.05 (m, 2H, C*H_2_*CH_2_COOH), 2.54 (t, 2H, CH_2_C*H_2_*COOH), 2.43 (s, 6H, 2CH_3_), 4.37 (t, 1H, NHC*H*COOH), 4.52 (s, 2H, pteridine-C*H*_2_-N), 6.45 (d, 2H, *J* = 8.4 Hz, benzene C_2_*H*,C_6_*H*), 6.68 (d, 2H, N-Ph-(*H*)_ortho_), 6.83 (d, 2H, *J* = 8.4 Hz, benzene C_3_*H*,C_5_*H*), 6.89 (s, 1H, *H*N-Ph), 7.61 (m, 2H, N-Ph-(*H*) _(meta)_), 8.13 (s, 1H, N*H*CO), 8.40 (t, 1H, pyrimidine C_3_*H*), 8.55 (s, 1H, pteridine-N*H*CONH), 8.61 (d, 2H, pyrimidine C_2_*H*,C_4_*H*), 8.79 (s,1H, pteridine-C_7_*H*), 8.91 (s, 1H, pteridine-NHCON*H*), 10.31 (s, 1H, pteridine N*H*), 11.31 (s, 1H, CH_2_CH_2_COO*H*), 12.34 (s,1H, CH_2_COO*H*), 12.85 (s, 1H, SO_2_N*H*). Anal. Calcd. for C_32_H_31_N_11_O_9_S (745.72): C, 51.54; H, 4.19; N, 20.66; S, 4.30; found: C, 51.21; H, 4.02; N, 20.49; S, 4.17.

#### 4.3.3. “*N*-[4-({[2-({[2-(aminocarbonyl)hydrazino]methylene}-amino)-4-oxo-3,4-dihydropteridin-6-yl]methyl}amino)benzoyl]glutamic acid” (**21**)

Compound **4** (0.001 mol, 0.5 g) and semicarbazide HCl (0.001 mol, 0.11 g) and drops of TMA in DMF (14 mL) was stirred under reflux for 4 h (TLC, *R_F_* = 0.65, eluent: CH_2_Cl_2_). Brown powder formed after crystallization from EtOH. Yield 78%, m.p. 250–252 °C. IR, 3412–3389 cm^−1^ (2OH), 3329–33251 cm^−1^ (NH_2_ and 5 NH), 2949 cm^−1^ (Ar-H), 2850 cm^−1^ (Aliphatic-H), 1666–1623 cm^−1^ (5C=O and C=N). ^1^H-NMR (DMSO *d_6_*, 850 MHz): δ = 1.91–2.05 (m, 2H, C*H_2_*CH_2_COOH), 2.54 (t, 2H, CH_2_C*H_2_*COOH), 4.31 (t, 1H, NHC*H*COOH), 4.44 (s, 2H, pteridine-C*H_2_*-N), 6.64 (d, 2H, N-Ph-(*H*)_ortho_), 6.89 (s, 1H, *H*N-Ph), 6.98 (s, 2H, NH_2_), 7.61 (m, 2H, N-Ph-(*H*) _(meta)_), 7.65 (s, 1H, N=C*H*), 8.21 (s, 1H, N*H*CO), 8.84 (s,1H, pteridine-C_7_*H*), 10.31 (s, 1H, pteridine N*H*), 10.57 (s, 1H, N*H*-NH), 10.99 (s, 1H, NH-N*H*), 11.41 (s, 1H, CH_2_CH_2_COO*H*), 12.28 (s,1H, CH_2_COO*H*). Anal. Calcd. for C_21_H_22_N_10_O_7_ (526.46): C, 47.91; H, 4.21; N, 26.61; found: C, 47.63; H, 3.92; N, 26.47.

#### 4.3.4. “*N*-{4-[({2-[({2-[amino(imino)methyl]hydrazino}-methylene)amino]-4-oxo-3,4-dihydropteridin-6-yl}methyl)amino]-benzoyl}glutamic acid” (**22**)

Compound **14** (0.001 mol, 0.5 g) and aminoguanidinium hydrocarbonate (0.001 mol, 0.14 g) in glacial AcOH (15 mL) was stirred under reflux (TLC, *R_F_* = 0.6, eluent: CH_2_Cl_2_), after 2 h a green precipitate formed, which crystallized from EtOH. Yield 96%, m.p. 278–280 °C. IR, 3442–3415 cm^−1^ (2OH), 3289–3151 cm^−1^ (NH_2_ and 6 NH), 2951 cm^−1^ (Ar-H), 2847 cm^−1^ (Aliphatic-H), 1672–1617 cm^−1^ (4C=O and C=N). ^1^H-NMR (DMSO *d_6_*, 850 MHz): δ = 1.93–2.05 (m, 2H, C*H_2_*CH_2_COOH), 2.52 (t, 2H, CH_2_C*H_2_*COOH), 4.29 (t, 1H, NHC*H*COOH), 4.51 (s, 2H, pteridine-C*H_2_*-N), 6.21 (s, 2H, NH_2_), 6.61 (d, 2H, N-Ph-(*H*)_ortho_), 6.90 (s, 1H, *H*N-Ph), 7.60 (m, 2H, N-Ph-(*H*) _(meta)_), 7.55 (s, 1H, N=C*H*), 7.82 (s, 1H, C=NH), 8.11 (s, 1H, N*H*CO), 8.82 (s,1H, pteridine-C_7_*H*), 10.13 (s, 1H, N*H*-NH), 10.33 (s, 1H, pteridine N*H*), 10.90 (s, 1H, NH-N*H*), 11.42 (s, 1H, CH_2_CH_2_COO*H*), 12.31 (s,1H, CH_2_COO*H*). Anal. Calcd. for C_21_H_23_N_11_O_6_ (525.48): C, 48.00; H, 4.41; N, 29.32; found: C, 47.61; H, 4.28; N, 29.10.

#### 4.3.5. “*N*-[4-({[2-({[(carboxymethyl)amino]methylene}amino)-4-oxo-3,4-dihydro-pteridin-6-yl]methyl}amino)benzoyl]glutamic acid” (**23**)

A mixture of compound **14** (0.001 mol, 0.5 g) and glycine (0.001 mol, 0.08 g) in DMF/ H_2_O mixture (9:1, 10 mL) refluxed for 3h (TLC, *R_F_* = 0.75, eluent: CH_2_Cl_2_). The brown solid formed crystallized from EtOH to give crystals. Yield, 91%, m.p. 218–220 °C. IR, 3443–3405 cm^−1^ (3OH), 3317–3122 cm^−1^ (4NH), 2910 cm^−1^ (Ar-H), 2850 cm^−1^ (Aliphatic-H), 1688–1619 cm^−1^ (5C=O and C=N). ^1^H-NMR (DMSO *d_6_*, 850 MHz): δ = 1.92–2.03 (m, 2H, C*H_2_*CH_2_COOH), 2.54 (t, 2H, CH_2_C*H_2_*COOH), 4.18 (s, 2H, NHC*H_2_*COOH), 4.33 (t, 1H, NHC*H*COOH), 4.55 (s, 2H, pteridine-C*H_2_*-N), 6.63 (d, 2H, N-Ph-(*H*)_ortho_), 6.90 (s, 1H, *H*N-Ph), 7.44 (m, 2H, N-Ph-(*H*) _(meta)_), 7.52 (s, 1H, N=C*H*), 8.09 (s, 1H, N*H*CO), 8.77 (s,1H, pteridine-C_7_*H*), 8.96 (s, 1H, N*H*CH_2_COOH), 10.36 (s, 1H, pteridine NH), 11.34 (s, 1H, CH_2_CH_2_COO*H*), 11.58 (s,1H, CH_2_COO*H*), 12.28 (s, 1H, NHCH_2_COO*H*). Anal. Calcd. for C_22_H_22_N_8_O_8_ (526.46): C, 50.19; H, 4.21; N, 21.28; found: C, 49.84; H, 4.06; N, 21.01.

### 4.4. Reaction of Compound ***14*** and Sulfa Drugs (***24a**,**b***): General Procedure

A mixture of **14** (0.001 mol, 0.5 g) and appropriate sulfa drug (0.001 mol) in DMF (15 mL) was stirred under reflux for 3h (TLC, *R_F_* = 0.4, eluent: CH_2_Cl_2_). The precipitate formed crystallized from EtOH to yield brownish powder.

#### 4.4.1. “*N*-(4-{[(4-oxo-2-{[({4-[(pyrimidin-2-ylamino)sulfonyl]-phenyl}amino) methylene]-amino}-3,4-dihydropteridin-6-yl)methyl]amino}-benzoyl)-glutamic acid” (**24a**)

Brownish powder, yield, 76%, m.p. 236–238 °C. IR, 3456–3412 cm^−1^ (2OH), 3321–3228 cm^−1^ (5NH), 2974 cm^−1^ (Ar-H), 2803 cm^−1^ (Aliphatic-H), 1687–1616 cm^−1^ (4C=O and C=N). ^1^H-NMR (DMSO *d_6_*, 850 MHz): δ = 1.93–2.05 (m, 2H, C*H_2_*CH_2_COOH), 2.53 (t, 2H, CH_2_C*H_2_*COOH), 4.32 (t, 1H, NHC*H*COOH), 4.52 (s, 2H, pteridine-C*H_2_*-N), 6.41 (d, 2H, *J* = 8.4 Hz, benzene C_2_*H*,C_6_*H*), 6.61 (d, 2H, N-Ph-(*H*)_ortho_), 6.84 (d, 2H, *J* = 8.4 Hz, benzene C_3_*H*,C_5_*H*), 6.91 (s, 1H, *H*N-Ph), 7.02 (s, 1H, N=CH-N*H*), 7.52 (s, 1H, N=C*H*), 7.64 (m, 2H, N-Ph-(*H*) _(meta)_), 8.11 (s, 1H, N*H*CO), 8.45 (t, 1H, pyrimidine C_3_*H*), 8.62 (d, 2H, pyrimidine C_2_*H*,C_4_*H*), 8.79 (s,1H, pteridine-C_7_*H*), 10.29 (s, 1H, pteridine NH), 11.44 (s, 1H, CH_2_CH_2_COO*H*), 12.28 (s,1H, CH_2_COO*H*), 12.87 (s, 1H, SO_2_N*H*). Anal. Calcd. for C_30_H_27_N_11_O_8_S (701.67): C, 51.35; H, 3.88; N, 21.96; S, 4.57; found: C, 51.02; H, 3.42; N, 21.71, S, 4.33.

#### 4.4.2. “*N*-[4-({[2-({[(4-{[(4,6-dimethylpyrimidin-2-yl)amino]-sulfonyl}phenyl)amino]-methylene}amino)-4-oxo-3,4-dihydropteridin-6-yl]methyl}-amino)benzoyl]glutamic acid” (**24b**)

Brown powder, yield, 74%, m.p. 258–260 °C. IR, 3453–3405 cm^−1^ (2OH), 3314–3212 cm^−1^ (5NH), 2952 cm^−1^ (Ar-H), 2851 cm^−1^ (Aliphatic-H), 1692–1621 cm^−1^ (4C=O and C=N). ^1^H-NMR (DMSO *d_6_*, 850 MHz): δ = 1.90–2.04 (m, 2H, C*H_2_*CH_2_COOH), 2.51 (t, 2H, CH_2_C*H_2_*COOH), 2.68 (s, 6H, 2CH_3_), 4. 30 (t, 1H, NHC*H*COOH), 4.52 (s, 2H, pteridine-C*H_2_*-N), 6.41 (d, 2H, *J* = 8.4 Hz, benzene C_2_*H*,C_6_*H*), 6.64 (d, 2H, N-Ph-(*H*)_ortho_), 6.80 (d, 2H, *J* = 8.4 Hz, benzene C_3_*H*,C_5_*H*), 6.91 (s, 1H, *H*N-Ph), 7.03 (s, 1H, N=CH-N*H*), 7.53 (s, 1H, N=C*H*), 7.61 (m, 2H, N-Ph-(*H*) _(meta)_), 8.11 (s, 1H, N*H*CO), 8.44 (t, 1H, pyrimidine C_3_*H*), 8.61 (d, 2H, pyrimidine C_2_*H*,C_4_*H*), 8.80 (s,1H, pteridine-C_7_*H*), 10.33 (s, 1H, pteridine NH), 11.45 (s, 1H, CH_2_CH_2_COO*H*), 12.31 (s,1H, CH_2_COO*H*), 12.84 (s, 1H, SO_2_N*H*). Anal. Calcd. for C_32_H_31_N_11_O_8_S (729.72): C, 52.67; H, 4.28; N, 21.11; S, 4.39; found: C, 52.40; H, 4.10; N, 21.03; S, 4.13.

#### 4.4.3. “*N*-(4-{[(4-amino-3-mercapto-2,12-dioxo-5-phenyl-1,12-dihydro-2H-pyrido[3′,2′:5,6]-pyrimido[2,1-b]pteridin-10-yl)methyl]-amino}benzoyl)-glutamic acid” (**25**)

Compound **10** (0.001 mol, 0.59 g), thioglycolic acid (0.001 mol, 0.09 g), and drops of TMA in EtOH (12 mL) were refluxed for 15 h (TLC, *R_F_* = 0.55, eluent: CH_2_Cl_2_). The precipitate formed crystallized from DMF/EtOH 1:1 to yield dark orange crystals. Yield, 76%, m.p. 251–253 °C. IR, 3401–3389 cm^−1^ (2OH), 3287–3165 cm^−1^ (NH_2_, 3NH), 2947 cm^−1^ (Ar-H), 2852 cm^−1^ (Aliphatic-H), 2657 cm^−1^ (SH), 1683–1622 cm^−1^ (5C=O and C=N). ^1^H-NMR (DMSO *d_6_*, 850 MHz): δ = 1.90–2.03 (m, 2H, C*H_2_*CH_2_COOH), 2.55 (t, 2H, CH_2_C*H_2_*COOH), 4.06 (t, 1H, NHC*H*COOH), 4.53 (s, 2H, pteridine-C*H_2_*-N), 6.92 (d, 2H, N-Ph-(*H*)_ortho_), 6.96 (s, 1H, *H*N-Ph), 7.68–7.90 (m, 7H, N-Ph-(*H*)_(meta)_ and pyrimidine-4-*Ph*), 8.12 (s, 1H, N*H*CO), 8.67 (s,1H, pteridine-C_7_*H*), 11.38 (s, 1H, pyridine-N*H*), 11.44 (s, 1H, CH_2_CH_2_COO*H*), 12.31 (s,1H, CH_2_COO*H*), 14.01 (s, 1H, pyridine-S*H*). Anal. Calcd. for C_31_H_25_N_9_O_7_S (667.65): C, 55.77; H, 3.77; N, 18.88; S, 4.80; found: C, 55.54; H, 3.62; N, 18.64; S, 4.67.

#### 4.4.4. “*N*-{4-[({8-cyano-9-[(mercaptoacetyl)amino]-11-oxo-7-phenyl-11H-pyrimido[2,1-b]pteridin-2-yl}methyl)amino]benzoyl}glutamic acid” (**26**)

Compound **10** (0.001 mol, 0.59 g) and thioglycolic acid (0.001 mol, 0.09 g) in EtOH (13 mL) were refluxed for 5 h (TLC, *R_F_* = 0.5, eluent: CH_2_Cl_2_). The precipitate formed on hot filtered and crystallized from DMF/EtOH 1:1 to produce yellowish brown crystals. Yield, 89%, m.p. 238–240 °C. IR, 3427–3412 cm^−1^ (2OH), 3248–3153 cm^−1^ (3NH), 2961 cm^−1^ (Ar-H), 2866 cm^−1^ (Aliphatic-H), 2634 cm^−1^ (SH), 2206 cm^−1^ (CN), 1678–1618 cm^−1^ (5C=O and C=N). ^1^H-NMR (DMSO *d_6_*, 850 MHz): δ = 1.91–2.07 (m, 2H, C*H_2_*CH_2_COOH), 2.53 (t, 2H, CH_2_C*H_2_*COOH), 3.64 (s, 2H, NHCOC*H*_2_SH), 4.06 (t, 1H, NHC*H*COOH), 4.52 (s, 2H, pteridine-C*H_2_*-N), 6.90 (d, 2H, N-Ph-(*H*)_ortho_), 6.99 (s, 1H, *H*N-Ph), 7.61-7.91 (m, 7H, N-Ph-(*H*)_(meta)_ and pyrimidine-4-*Ph*), 8.19 (s, 1H, N*H*CO), 8.66 (s,1H, pteridine-C_7_*H*), 10.37 (s, 1H, N*H*COCH_2_SH), 11.44 (s, 1H, CH_2_CH_2_COO*H*), 12.28 (s,1H, CH_2_COO*H*), 13.97 (s, 1H, NHCOCH_2_S*H*). Anal. Calcd. for C_31_H_25_N_9_O_7_S (667.65): C, 55.77; H, 3.77; N, 18.88; S, 4.80; found: C, 55.54; H, 3.62; N, 18.64; S, 4.67.

#### 4.4.5. “*N*-{4-[({8-cyano-9-[(mercaptocarbonothioyl)amino]-11-oxo-7-phenyl-11H-pyrimido-[2,1-b]pteridin-2-yl}methyl)amino]benzoyl}-glutamic acid” (**27**)

Compound **10** (0.001 mol, 0.59 g), CS_2_ (excess, 1 mL) and KOH (0.003 mol, 0.17 g) in EtOH (20 mL) was refluxed for 5 h (TLC, *R_F_* = 0.35, eluent: CH_2_Cl_2_). The solution poured onto crushed ice after concentration then acidified with dilute HCl. The precipitate formed crystallized from EtOH to produce reddish brown powder. Yield, 57%, m.p. 240–242 °C. IR, 3418–3406 cm^−1^ (2OH), 3221–3170 cm^−1^ (3NH), 2950 cm^−1^ (Ar-H), 2838 cm^−1^ (Aliphatic-H), 2630 cm^−1^ (SH), 2202 cm^−1^ (CN), 1689–1621 cm^−1^ (4C=O and C=N), 1328 cm^−1^ (C=S). ^1^H-NMR (DMSO *d_6_*, 850 MHz): δ = 1.92–2.04 (m, 2H, C*H_2_*CH_2_COOH), 2.54 (t, 2H, CH_2_C*H_2_*COOH), 4.05 (t, 1H, NHC*H*COOH), 4.41 (s, 2H, pteridine-C*H_2_*-N), 6.90 (d, 2H, N-Ph-(*H*)_ortho_), 6.95 (s, 1H, *H*N-Ph), 7.61-7.95 (m, 7H, N-Ph-(*H*) _(meta)_ and pyrimidine-4-*Ph*), 8.18 (s, 1H, N*H*CO), 8.66 (s,1H, pteridine-C_7_*H*), 10.56 (s, 1H, N*H*C=S); 11.41 (s, 1H, CH_2_CH_2_COO*H*), 12.29 (s,1H, CH_2_COO*H*), 13.98 (s, 1H, S*H*). Calcd. for C_30_H_23_N_9_O_6_S_2_ (669.69): C, 53.80; H, 3.46; N, 18.82; S, 9.58; found: C, 53.66; H, 3.39; N, 18.71; S, 9.42.

#### 4.4.6. “*N*-(4-{[(12-oxo-5-phenyl-2,4-dithioxo-1,3,4,12-tetrahydro-2H-pyrimido-[5′,4′:5,6]-pyrimido[2,1-b]pteridin-10-yl)methyl]- amino}benzoyl)glutamic acid” (**28**)

Compound **10** (0.001 mol, 0.59 g), CS_2_ (excess, 1 mL) and KOH (0.003 mol, 0.17 g, 3 mmol) dry pyridine (10 mL) was refluxed for 12 h (TLC, *R_F_* = 0.5, eluent: CH_2_Cl_2_). The solution poured onto crushed ice/dilute HCl. The product crystallized from EtOH to yield orange powder. Yield, 57%, m.p. 262–264 °C. IR, 3419–3410 cm^−1^ (2OH), 3234–3187 cm^−1^ (4NH), 2971 cm^−1^ (Ar-H), 2846 cm^−1^ (Aliphatic-H), 1675–1623 cm^−1^ (4C=O and C=N); 1350–1273 (2C=S). ^1^H-NMR (DMSO *d_6_*, 850 MHz): δ = 1.93–2.05 (m, 2H, C*H_2_*CH_2_COOH), 2.54 (t, 2H, CH_2_C*H_2_*COOH), 4.01 (t, 1H, NHC*H*COOH), 4.44 (s, 2H, pteridine-C*H_2_*-N), 6.91 (d, 2H, N-Ph-(*H*)_ortho_), 6.97 (s, 1H, *H*N-Ph), 7.62–7.90 (m, 7H, N-Ph-(*H*) _(meta)_ and pyrimidine-4-*Ph*), 8.22 (s, 1H, N*H*CO), 8.67 (s,1H, pteridine-C_7_*H*), 10.56 (s, 1H, N_1_*H*C=S); 11.41 (s, 1H, CH_2_CH_2_COO*H*), 12.29 (s,1H, CH_2_COO*H*), 12.56 (s, 1H, N_2_*H*C=S). Calcd. for C_30_H_23_N_9_O_6_S_2_ (669.69): C, 53.80; H, 3.46; N, 18.82; S, 9.58; found: C, 53.66; H, 3.39; N, 18.71; S, 9.42.

#### 4.4.7. “*N*-(4-{[(2,4-diamino-3-cyano-12-oxo-5-phenyl-12H-pyrido[3′,2′:5,6]pyrimido[2,1-b]pteridin-10-yl)methyl]amino}benzoyl)-glutamic acid” (**29**)

Compound **10** (0.001 mol, 0.59 g) with malononitrile (0.001 mol, 0.07 g), and drops of TMA in EtOH (13 mL) refluxed for 13 h (TLC, *R_F_* = 0.6, eluent: CH_2_Cl_2_). The red product formed crystallized from EtOH to produce dark red powder. Yield, 69%, m.p. 236–238 °C. IR, 3423–3415 cm^−1^ (2OH), 3245–3162 cm^−1^ (2NH_2_, 2NH), 2944 cm^−1^ (Ar-H), 2852 cm^−1^ (Aliphatic-H), 2201 cm^−1^ (CN); 1678–1621 cm^−1^ (4C=O and C=N). ^1^H-NMR (DMSO *d_6_*, 850 MHz): δ = 1.93–2.04 (m, 2H, C*H_2_*CH_2_COOH), 2.55 (t, 2H, CH_2_C*H_2_*COOH), 4.11 (t, 1H, NHC*H*COOH), 4.42 (s, 2H, pteridine-C*H_2_*-N), 6.12 (s, 2H, *C_2_*N*H*_2_), 6.58 (s, 2H, *C_4_*N*H*_2_), 6.92 (d, 2H, N-Ph-(*H*)_ortho_), 6.99 (s, 1H, *H*N-Ph), 7.62–7.93 (m, 7H, N-Ph-(*H*) _(meta)_ and pyrimidine-4-*Ph*), 8.21 (s, 1H, N*H*CO), 8.61 (s,1H, pteridine-C_7_*H*), 11.43 (s, 1H, CH_2_CH_2_COO*H*), 12.22 (s,1H, CH_2_COO*H*). Calcd. for C_32_H_25_N_11_O_6_ (659.61): C, 58.27; H, 3.82; N, 23.36; found: C, 58.05; H, 3.71; N, 23.23.

#### 4.4.8. “*N*-(4-{[(4-amino-12-oxo-5-phenyl-12H-pyrimido-[5′,4′:5,6]pyrimido-[2,1-b]pteridin-10-yl)methyl]amino}benzoyl)glutamic acid” (**30**)

Compound **10** (0.001 mol, 0.59 g) refluxed in excess formamide (8 mL) for 9 h (TLC, *R_F_* = 0.7, eluent: CH_2_Cl_2_). The solution poured on ice-cold water and then extracted with CH_2_Cl_2_ (25 mL, two times) to give yellow product which crystallized from EtOH. Yield, 42%, m.p. 225–227 °C. IR, 3421–3411 cm^−1^ (2OH), 3221–3137 cm^−1^ (NH_2_, 2NH), 2957 cm^−1^ (Ar-H), 2850 cm^−1^ (Aliphatic-H), 1671–1623 cm^−1^ (4C=O and C=N). ^1^H-NMR (DMSO *d_6_*, 850 MHz): δ = 1.88–2.05 (m, 2H, C*H_2_*CH_2_COOH), 2.50 (t, 2H, CH_2_C*H_2_*COOH), 4.07 (t, 1H, NHC*H*COOH), 4.45 (s, 2H, pteridine-C*H_2_*-N), 6.50 (s, 2H, *C_4_*N*H*_2_), 6.90 (d, 2H, N-Ph-(*H*)_ortho_), 6.98 (s, 1H, *H*N-Ph), 7.60–7.97 (m, 7H, N-Ph-(*H*) _(meta)_ and pyrimidine-4-*Ph*, pyrimidine C_2_*H*), 8.23 (s, 1H, N*H*CO), 8.68 (s,1H, pteridine-C_7_*H*), 11.39 (s, 1H, CH_2_CH_2_COO*H*), 12.28 (s,1H, CH_2_COO*H*). Calcd. for C_30_H_24_N_10_O_6_ (620.57): C, 58.06; H, 3.90; N, 22.57; found: C, 57.83; H, 3.76; N, 22.50.

### 4.5. Biological Activity (Sensitivity Tests) by Kirby–Bauer Method

Antimicrobial activity of the tested samples was determined using a modified Kirby–Bauer disc diffusion method [39,40,41,42,43,44].

## Figures and Tables

**Table 1:** Biological activity of some new synthesized compounds.

Sample		Inhibition Zone Diameter (mm/mg Sample)					
		Bacterial Species				Fungi	
		(G^+^)		(G^−^)			
		*B. subtilis*	*S. aureus*	*E. coli*	*P. aeruginosa*	*A. flavus*	*C. albicans*
Control: DMSO		0.0	0.0	0.0	0.0	0.0	0.0
Standard	Ampicillin Antibacterial agent	26	21	25	26	--	--
	Amphotericin B Antifungal agent	--	--	--	--	17	21
**Folic acid 1**		10	10	10	10	0.0	0.0
**2**		10	9	10	11	0.0	0.0
**4**		9	9	9	9	0.0	0.0
**5**		0.0	0.0	0.0	0.0	0.0	0.0
**6a**		12	12	12	13	0.0	0.0
**8**		12	12	13	13	0.0	0.0
**9**		10	0.0	0.0	12	0.0	0.0
**10**		10	9	9	10	0.0	0.0
**12**		9	10	12	12	0.0	0.0
**14**		10	10	10	10	0.0	0.0
**15**		10	10	11	10	0.0	0.0
**18**		0.0	0.0	0.0	0.0	0.0	0.0
**20b**		0.0	9	10	0.0	0.0	0.0
**22**		13	12	12	12	0.0	0.0
**23**		9	10	10	10	0.0	0.0
**24b**		10	9	10	10	0.0	0.0
**27**		11	12	11	14	0.0	0.0

G: Gram reaction. Solvent: DMSO.

## Supplementary Materials

The following are available online Spectral data as Supplementary Materials.

## Sample Availability

Samples of the compounds are available from the authors.

## References

[1] National Center for Biotechnology Information PubChem Compound Database; CID¼6037. https://pubchem.ncbi.nlm.nih.gov/compound/6037.

[2] Herbert V., Zalusky R. (1962). Interrelations of vitamin B12 and folic acid metabolism: Folic acid clearance studies. J. Clin. Investig..

[3] Quinlivan E.P., McPartlin J., McNulty H., Ward M., Strain J.J., Weir D.G., Scott J.M. (2002). Importance of both folic acid and vitamin B12 in reduction of risk of vascular disease. Lancet.

[4] Pitkin R.M. (2007). Folate and neural tube defects. Am. J. Clin. Nutr..

[5] Green N.S. (2002). Folic Acid Supplementation and Prevention of Birth Defects. J. Nutr..

[6] Duthie S.J. (1999). Folic acid deficiency and cancer: Mechanisms of DNA instability. Br. Med. Bull..

[7] Aleem G., Yibin Z., Mc John J., Roy L.K. (2002). Synthesis of Classical and Nonclassical Partially Restricted Linear Tricyclic 5-Deaza Antifolates. J. Med. Chem..

[8] Edward C.T., Jerauld S.S., Stephen R.F. (1985). Synthesis of DL-7,10-ethano-5-deazaaminopterin and L-7,10-ethano-5-deazafolic acid. J. Org. Chem..

[9] Joseph I.D., Pamela H.C., Roy L.K., Yvette G., Francis M.S. (1990). Synthesis and antifolate properties of 10-alkyl-5,10-dideaza analogs of methotrexate and tetrahydrofolic acid. J. Med. Chem..

[10] Kotake Y., Okauchi T., Iijima A., Yoshimatsu K., Nomura H. (1995). Novel 6-5 fused ring heterocycle antifolates with potent antitumor activity: Bridge modifications and heterocyclic benzoyl isosters of 2,4-diamino-6,7-dihydro-5*H*-cyclopenta[*d*]pyrimidine antifolate. Chem. Pharm. Bull. (Tokyo).

[11] Andre R., Henry B., Joel E.W., Richard G.M. (1994). 5-Deaza-7-desmethylene analogues of 510-methylene-5678-tetrahydrofolic acid and related compounds: Synthesis and in vitro biological activity. J. Heterocycl. Chem..

[12] Bailey L.B., Rampersaud G.C., Kauwell G.P.A. (2003). Folic Acid Supplements and Fortification Affect the Risk for Neural Tube Defects, Vascular Disease and Cancer: Evolving Science. J. Nutr..

[13] Arzeni C., Pérez O.E., LeBlanc J.G., Pilosof A.M.R. (2015). Egg albumin–folic acid nanocomplexes: Performance as a functional ingredient and biological activity. J. Funct. Foods.

[14] Kronenberg G., Colla M., Endres M. (2009). Folic acid, neurodegenerative and neuropsychiatric disease. Curr. Mol. Med..

[15] Mao X., Xing X., Xu R., Gong Q., He Y., Li S., Wang H., Liu C., Ding X., Na R. (2016). Folic acid and vitamins D and B12 correlate with homocysteine in Chinese patients with type-2 diabetes mellitus, hypertension, or cardiovascular disease. Medicine.

[16] Keser I., Ilich J.Z., Vrkic N., Giljevic Z., Colic Baric I. (2013). Folic acid and vitamin B_12_ supplementation lowers plasma homocysteine but has no effect on serum bone turnover markers in elderly women: A randomized, double-blind, placebo-controlled trial. Nutr. Res..

[17] Solini A., Santini E., Ferrannini E. (2006). Effect of short-term folic acid supplementation on insulin sensitivity and inflammatory markers in overweight subjects. Int. J. Obes..

[18] Moens A.L., Champion H.C., Claeys M.J., Tavazzi B., Kaminski P.M., Wolin M.S., Borgonjon D.J., van Nassauw L., Haile A., Zviman M. (2008). High-dose folic acid pretreatment blunts cardiac dysfunction during ischemia coupled to maintenance of high-energy phosphates and reduces postreperfusion injury. Circulation.

[19] Saad H. (1996). Synthesis of some pyridyloxymethyloxadiazoles, thiazoles and triazoles of expected pharmacological activity. Indian J. Chem..

[20] Saad H.A., Allimony H.A., El-Mariah F.A.A. (1998). Synthesis and Antimicrobial Activity of Some Nitrogen Heterobicyclic Systems: Part II. Indian J. Chem..

[21] Allimony H.A., Saad H.A., El-Mariah F.A.A. (1999). Synthesis and antimicrobial activity of some nitrogen heterobicyclic systems: Part I. Indian J. Chem. Sec. B Org. Med. Chem..

[22] El-Mariah F.A.A., Saad H.A., Allimony H.A., Abdel-Rahman R.M. (2000). Synthesis and Antimicrobial Activity of Some Nitrogen Heterobicyclic Systems: Part III. Indian J. Chem..

[23] Saad H.A., Moustafa H.Y., Assy M.G., Sayed M.A. (2001). Functionlization and Heteroannelation of Ethyl 2-(4′-chlorophenyl)-4-mercapto-6-methylpyrimidine-5-carboxylate. Bull. Korean Chem. Soc..

[24] Saad H.A., Mokbil M.N., El-Gendy A.M., Haikal A.Z. (2002). Synthesis of some glycosides of pyridinone derivatives. AFINIDAD.

[25] Deeb A., Saad H. (2003). Tetrazolo[1,5-*b*]pyridazine-8-carbohydrazide, Synthesis and some reactions. Heterocycles.

[26] Moustafa A.H., Saad H.A. (2005). Synthesis of Phenazone Derivatives. J. Chem. Res..

[27] Saad H.A., Moustafa A.H. (2006). Synthesis of Fused heteropolycyclic systems containing an indole moiety. J. Chem. Res..

[28] Moustafa A.H., Saad H.A., Shehab W.S., El-Mobayed M.M. (2008). Synthesis of Some New Pyrimidine Derivatives of Expected Antimicrobial Activity. Phosphorus Sulfur Silicon Relat. Elem..

[29] Saad H.A., Youssef M.M., Mosselhi M.A. (2011). Microwave Assisted Synthesis of Some New Fused 1,2,4-Triazines Bearing Thiophene Moieties with Expected Pharmacological Activity. Molecules.

[30] Saad H.A., Moustafa A.H. (2011). Synthesis and Anticancer Activity of Some New *S*-Glycosyl and S-Alkyl 1,2,4-Triazinone Derivatives. Molecules.

[31] Saad H.A. (2012). Synthesis of novel fused heterocyclic compounds from 7-amino-[1,2,4]triazino[3,4-*b*][1,3,4]thiadiazine-8-carbonitrile. Curr. Org. Synth..

[32] Aly M.R.E., Gobouri A.A., Abdel Hafez S.H., Saad H.A. (2015). Synthesis Reactions and Biological Activity of some Triazine Derivatives Containing Sulfa Drug Moieties. Russ. J. Bioorg. Chem..

[33] Aly M.R.E., Saad H.A., Abdel Hafez S.H. (2015). Three-Component Process for the Synthesis of Pyrimido[2,1-*c*][1,2,4]triazine Derivatives via Knoevenagel Condensation under Thermal Aqueous Conditions. Curr. Org. Synth..

[34] Aly M.R.E., Saad H.A., Mohamed M.A.M. (2015). Click reaction based synthesis, antimicrobial, and cytotoxic activities of new 1,2,3-triazoles. Bioorg. Med. Chem. Lett..

[35] Aly M.R.E., Saad H.A., Abdel Hafez S.H. (2015). Synthesis, antimicrobial and cytotoxicity evaluation of new cholesterol congeners. Beilstein J. Org. Chem..

[36] Amin M.A., Saad H.A. (2016). Synthesis and Biological Activity of Fused Heteropolycyclic Systems Containing an Indole Moiety. Curr. Org. Synth..

[37] Abdel-Rahmana R.M., Saad H.A. (2016). Synthesis and Chemical Behavior of 1,2,4-Triazine Derivatives Bearing/Containing Phosphorus Amides Donor-Accepto Interactions—A Review. Curr. Org. Synth..

[38] El Azab I.H., Saad H.A. (2016). Thiosemicarbazides, Potent Reagent for Synthesis of Some New 1,4-Diphenylbenzo[g]quinoxaline-5,10-Dione Based Heterocycles. Heterocycles.

[39] AlHarthi R.R., Ali R.S., Amina M.A., Saad H.A. (2016). Synthesis of Some New Fused 1,2,4-Triazines of Expected Antimicrobial Activity. Russ. J. Gen. Chem..

[40] Shehab W.S., Saad H.A., Mouneir S.M. (2017). Synthesis and Antitumor/Antiviral Evaluation of 6–Thienyl–5–cyano-2– thiouracil Derivatives and Their Thiogalactosides Analogs. Curr. Org. Synth..

[41] Mohamed M.A.M., Abu-Alola L.M.B., Al-Zaidi O.N.A., Saad H.A.H. (2017). Cyclocondensation Reactions of Hydrazonoyl Chlorides with Some Azines: Synthesis of New Fused Heterocycles of Expected Microbiological Activity. Int. J. Org. Chem..

[42] Ali R.S., Saad H.A. (2018). Synthesis of Some Novel Fused Pyrimido[4″,5″:5′,6′][1,2,4]triazino-[3′,4′:3,4][1,2,4]triazino[5,6-*b*]indoles with Expected Anticancer Activity. Molecules.

[43] Ali R.S., Saad H.A. (2018). Synthesis and Pharmacological Studies of Unprecedented Fused Pyridazino[3′,4′:5,6][1,2,4]triazino[3,4-*b*][1,3,4]-thiadiazine Derivatives. Molecules.

[44] Bauer A.W., Kirby W.M., Sherris C., Turck M. (1966). Antibiotic susceptibility testing by a standardized single disk method. Am. J. Clin. Pathol..

[45] Pfaller M.A., Burmeister L., Bartlett M.A., Rinaldi M.G. (1988). Multicenter evaluation of four methods of yeast inoculum preparation. J. Clin. Microbiol..

[46] National Committee for Clinical Laboratory Standards (1997). Performance Antimicrobial Susceptibility of Flavobacteria.

[47] National Committee for Clinical Laboratory Standards (1993). Methods for Dilution Antimicrobial Susceptibility Tests for Bacteria that Grow Aerobically. Approved Standard M7-A3.

[48] Liebowitz L.D., Ashbee H.R., Evans E.G.V., Chong Y., Mallatova N., Zaidi M., Gibbs D., Global Antifungal Surveillance Group (2001). A two-year global evaluation of the susceptibility of Candida species to fluconazole by disk diffusion. Diagn. Microbiol. Infect. Dis..

[49] Matar M.J., Ostrosky-Zeichner L., Paetznick V.L., Rodriguez J.R., Chen E., Rex J.H. (2003). Correlation between E-test, disk diffusion, and microdilution methods for antifungal susceptibility testing of fluconazole and voriconazole. Antimicrob. Agents Chemother..

